# Quantifying cognitive effort’s impact on suppression of epilepsy-associated after discharges

**DOI:** 10.3389/fnetp.2026.1768476

**Published:** 2026-05-19

**Authors:** Sai Pavan Beeram, Matthew Farris, Samir Hossain, Nicholas Rethans, Joon Y. Kang, Emily A. Pereira

**Affiliations:** 1 Department of Electrical and Computer Engineering, Texas Tech University, Lubbock, TX, United States; 2 Department of Neurology, Johns Hopkins University, Baltimore, MD, United States

**Keywords:** brain-state modulation, cognitive effort, detrended fluctuation analysis, epilepsy, fractional-order dynamical networks, intracranial EEG, multifractal DFA, network physiology

## Abstract

Epilepsy affects over 50 million people worldwide, with approximately 30% of patients experiencing seizures that are resistant to medication, significantly impacting their quality of life. In this study, we analyze a unique dataset of intracranial electroencephalography (iEEG) recordings from patients undergoing functional brain mapping prior to epilepsy surgery. During this procedure, electrodes are implanted to identify critical brain regions, and brief pulse electrical stimulation is applied. Often, abnormal brain activity, known as epilepsy-associated after discharges (EAADs), are triggered by stimulation. During these events, patients are engaged in cognitive tasks to exert mental effort, such as solving arithmetic and spelling problems, as a potential method to suppress EAADs. We present evidence that exerting mental effort to perform cognitive tasks can suppress EAADs. Using detrended fluctuation analysis (DFA), multifractal DFA (MFDFA), and fractional-order dynamical network models (FODN), we characterize the temporal and spatial dynamics of iEEG data during these events to identify conditions and classify the trials under which cognitive effort is most effective to suppress EAADs. Our logistic regression model achieves an average accuracy of 77% using leave-one-out cross-validation. Our findings pave the way for a promising, non-invasive therapeutic avenue for managing epileptic activity through targeted cognitive activation. Our work lays the groundwork for novel brain-state modulation strategies for the treatment of epilepsy.

## 1 Introduction

The human brain is a complex high-dimensional physiological network (Ivanov, 2021) that can be adversely affected by neurological diseases, including epilepsy. Recent work has shown that epileptic brain signals, as measured by intracranial EEG, possesses long-range memory dependence (Cook et al., 2014), meaning that the data follows a power-law relationship, where the power-law exponent, known as the Hurst exponent, is computed using detrended fluctuation analysis (DFA). Hence, the Hurst exponent quantifies the multi-scale temporal nature of brain data (Hardstone et al., 2012), providing a framework to study neural dynamics across scales.

When iEEG data exhibits multi-fractal properties, meaning that it follows multiple power-law relationships over different time scales, the generalized Hurst exponent is computed using multifractal detrended fluctuation analysis (MFDFA) (Kantelhardt et al., 2002). MFDFA characterizes how fractal properties vary across different time scales, allowing us to identify subtle, nonstationary signatures that may not be detectable through single-scale measurements alone (Ihlen, 2012). Recent work showed that epileptic brain signals, as measured by intracranial EEG, exhibit multi-fractal temporal properties (John et al., 2025).

Previous studies have identified fractal properties in iEEG epileptic seizure data using multivariate eigen-wavelet analysis (Lucas et al., 2023), generalized Hurst exponents to distinguish healthy from epileptic activity (Lahmiri, 2018), and fractal features for machine learning classification in a clinical context (França et al., 2018). Hence, the Hurst exponent and generalized Hurst exponent characterize the fractal and multi-fractal temporal nature of epileptic iEEG data, respectively.

While epileptic brain signals exhibit fractal patterns, they also exhibit changing network interactions over time (Bassett and Sporns, 2017). To capture these changes in network interactions over time, fractional dynamical network models (FODN) have been proposed (Reed et al., 2022), which provide a spatially resolved picture of neural dynamics by quantifying influence from electrodes on other electrodes. FODN models not only capture the spatial dependence but also capture the long-range temporal dependence using a wavelet-based approach (Flandrin, 2002). Recent work has shown that the stability and multi-scale properties change and exhibit distinct patterns over epileptic iEEG recordings (Wang et al., 2025) as measured by the eigenvalues and fractional-order exponents of FODN models. Hence, FODN models capture the spatial-temporal relationships present in intracranial EEG data.

In this work, we study intracranial electroencephalography (iEEG) data collected from 15 epileptic patients undergoing pre-surgical evaluation. Patients received electrical stimulation to localize functional regions that should not be disturbed during surgery. During stimulation, EAADs sometimes appeared on the iEEG. During these events, the patient was asked arithmetic or spelling questions to investigate whether exerting cognitive effort to complete these tasks helped to suppress EAADs. Cognitive effort successfully suppressed EAADs in 50/116 (43.1%) of these trials (Lesser et al., 2019). In this work, we compute and analyze the Hurst exponents, generalized Hurst exponents, and eigenvectors of FODN models from iEEG during successful and failed trials to evaluate how cognitive effort influences fractal properties and network interactions across time scales to effectively suppress EAADs. Recent work has accurately characterized different cognitive tasks using generalized Hurst exponents computed with MFDFA from EEG data, including meditation and attention tasks (Wang, 2025) as well as perception and attention tasks (Gaurav et al., 2021). Our results provide evidence that our spatiotemporal framework captures important properties of the data to identify when cognitive effort will effectively suppress EAADs and when cognitive effort is unable to suppress EAADs.

Preliminary results in (Muldoon et al., 2018; Lesser et al., 2019; Lesser et al., 2022; Lesser et al., 2021; Lesser et al., 2023; Lesser et al., 2024) suggested that cognitive effort (i.e., exerting mental effort to answer arithmetic and spelling questions) suppresses epilepsy-associated abnormal spiking activity, known as after discharges (EAADs). These studies have experimented with different metrics to explain the underlying mechanisms behind the success of cognitive effort in suppressing EAADs from iEEG data. Initially, the work in (Muldoon et al., 2018) suggested that locally stable brain regions (computed using functional connectivity) may be an indicator as to whether EAADs would stop during cognitive effort. The work in (Lesser et al., 2019) found that during cognitive effort in successful trials there was a decrease in coherence across the brain in the 7.13–22.53 Hz frequency ranges as measured by wavelet-cross coherence, suggesting that the whole brain changes during EAADs suppression and not just a particular brain region. An extended study (Lesser et al., 2022) also using wavelet-cross coherence found a similar result regardless of the combination of electrodes being computed across the brain, suggesting the idea of a pan-cortical effect during cognitive effort and suppression of EAADs. Later, it was suggested that attention may play a role along with cognitive effort as the driver behind EAADs suppression (Lesser et al., 2021; Lesser et al., 2023; Lesser et al., 2024) based on the timing of the questions, the responses, and the suppression of these discharges. These studies have mostly studied this data using only wavelet-cross correlation (Lesser et al., 2019; Lesser et al., 2022; Lesser et al., 2023; Lesser et al., 2024), which is unable to capture the multi-scale structure of the data (Betzel and Bassett, 2017) and quantify network changes over time (Medaglia et al., 2015).

Alternative non-invasive methods for suppressing epileptic seizures in medically refractory epileptic patients are needed to guarantee seizure freedom and ensure a better quality of life for these patients. Our work provides a framework to understand how cognitive effort may be used as a future non-invasive method to suppress epileptic activity.

To our knowledge, our study is the first to quantify the impact of cognitive effort, during arithmetic and spelling tasks, on epileptic activity suppression in iEEG data by analyzing the Hurst exponents, generalized Hurst exponents, or fractional-order dynamical networks.

## 2 Methods

### 2.1 Intracranial EEG data

Using a Stellate Harmonie (Natus Medical Incorporated, Pleasanton, CA, United States of America) system that was capable of recording up to 128 channels with 1000 samples per second per channel along with a Schwarzer EEG Amplifiers Model 210,033 (Natus Europe GmbH, Robert-Koch-Str. 1, Planegg, Germany), intracranial EEG (iEEG) recordings were collected from 15 epileptic patients undergoing pre-surgical evaluation. These recordings were obtained as part of a clinical presurgical monitoring procedure and were not sourced from publicly available datasets. The data were collected at Johns Hopkins University under a study protocol approved by the Institutional Review Board (IRB) and were made available for this study through collaboration with Dr. Joon Y. Kang (Department of Neurology, Johns Hopkins University, Baltimore, MD, United States of America). All data were de-identified prior to analysis. Informed consent was obtained from participants or waived by the IRB in accordance with institutional guidelines. These patients received electrical stimulation to localize functional regions. The dataset consists of recordings from 15 patients, with stimulation applied at different cortical locations across subjects, resulting in variability in spatial activation patterns and network responses. This variability allows us to evaluate the robustness of the proposed framework across different stimulation conditions. During these clinical stimulation trials, cognitive tasks (e.g., arithmetic and spelling) were used as a means to suppress EAADs that sometimes occurred following stimulation. The cognitive tasks were selected because they are commonly used in clinical functional mapping and are known to engage distributed cortical networks, including language and numerical processing regions (Dehaene et al., 2005; Rapp and Lipka, 2011). These tasks require sustained cognitive effort, making them suitable for investigating the role of cognitive engagement in modulating EAADs. Table 1 shows the clinical data for all epileptic patients in our preliminary data set. Noise-dominated electrodes and other artifact channels identified during preprocessing were removed to avoid assessing effects from non-physiological data. Due to inconsistencies in behavioral time stamps, only 11 of the 15 subjects were retained for analysis.

**TABLE 1:** Clinical data for epileptic patients who participated in cognitive suppression trials.

Patient	Gender	Age onset	Seizure type	Electrodes	Total trials	Total success
1	F	21y	FA FI	86	18	9
2	M	16y	FA FB	73	3	2
3	F	3m	FA	82	3	3
4	M	10y	FA FI	56	2	0
5	M	8h	FA FI FB	75	2	2
6	M	46y	FA FI FB	91	1	1
7	F	10y	FA FB	99	2	1
8	F	7y	FA FI FB	59	2	0
9	F	12y	FI FB	79	1	1
10	M	Childhood	FA FB	109	2	0
12	F	47y	FA FI	105	1	1
13	F	13y	FI	80	1	0
14	M	8y	FI	88	4	0
15	F	15y	FI	110	1	0

**FIGURE 1:**
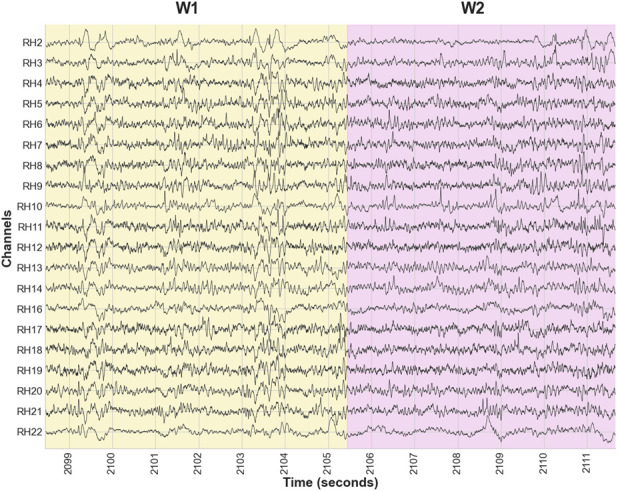
Intracranial EEG recordings from Patient 2, Trial 7, showing a subset of 20 channels. Window W1 starts at the time of electrical discharges and ends before questioning; W2 starts at the time of questioning and ends when the electrical discharges end.

Figure 1 shows a subset of 20 intracranial EEG (iEEG) channels for Patient 1, Trial 6, normalized (z-scored) across time for visualization. For each trial, the iEEG data were segmented into W1 , which started from the moment when the EAADs began to the moment when the patient was asked a arithmetic or spelling question, and W2 , which continued from the time of questioning until the moment when the EAADs either were suppressed (indicating a successful trail) or not suppressed (indicating a failed trail).

### 2.2 Detrended fluctuation analysis

Detrended Fluctuation Analysis (DFA) is a method for quantifying the presence of multi-scale fluctuations in a signal (Ihlen, 2012; Hardstone et al., 2012; Kantelhardt, 2008). Given an integrated signal

$$Y\left(i\right)=\overset{i}{\underset{k=1}{\sum }}\left[x\left(k\right)-\bar{x}\right],$$

where x(k)∈R is a signal, k∈{1,…,N} , and $\bar{x}$ is mean of x , the integrated time series Y is divided into $N_{s}=\text{int}\left(N/s\right)$ non-overlapping segments of length $s=\left\{2^{4},2^{5},2^{6},2^{7},\ldots \right\}$ . In each segment $\nu =\left\{1,\ldots ,N_{s}\right\}$ , a local polynomial trend $P_{\nu }\left(i\right)$ is removed, and the detrended variance is computed as

$$F^{2}\left(\nu ,s\right)=\frac{1}{s}\overset{s}{\underset{i=1}{\sum }}{\left[Y\left(\left(\nu -1\right)s+i\right)-P_{\nu }\left(i\right)\right]}^{2}.$$

In standard DFA (monofractal DFA), the fluctuation function is computed as the root-mean-square (RMS) of these detrended variances:

$$F\left(s\right)=\sqrt{\frac{1}{N_{s}}\overset{N_{s}}{\underset{\nu =1}{\sum }}F^{2}\left(\nu ,s\right)}.$$

The scaling relationship

$$F\left(s\right)∼s^{H}$$

is then used to estimate the Hurst exponent H , which characterizes long-range dependence in the signal. The Hurst exponent reflects the degree of scale invariance in the temporal dynamics: signals with higher H exhibit more persistent, self-similar fluctuations whose statistical structure follows a power-law across multiple time scales (Kantelhardt, 2008; Ihlen, 2012).

In this study, we applied DFA directly to preprocessed iEEG time series that were z-scored and cleaned of artifact-dominated channels, rather than to band-limited signals or amplitude envelopes. We used a first-order (linear) polynomial detrending within each segment. We estimated the scaling exponent over a range of window sizes corresponding to $s\in \left[2^{4},2^{5},2^{6},2^{7}\right]$ , which is appropriate for the short-duration (500 ms) segments analyzed in this study. We did not observe clear crossover behavior within this scaling range. We note that the choice of preprocessing, detrending order, and scaling range can influence DFA estimates, particularly for nonstationary physiological signals, as discussed in prior studies (Hu et al., 2001; Chen et al., 2005; Xu et al., 2005). We computed the Hurst exponent of every channel in 500 ms time windows for each trial. In addition to the original non-overlapping windows, we also used a 50% overlapped scheme, −0.5 s windows advanced every 0.25 s, to increase temporal resolution while acknowledging that adjacent estimates are partially dependent. We compared the distribution of the Hurst exponents in W1 against the distribution of the Hurst exponents in W2 to quantify the impact of cognitive effort. More specifically, we compared the Hurst exponent distributions for W1 and W2 channel by channel using the Kolmogorov–Smirnov test.

### 2.3 Multifractal detrended fluctuation analysis

Multifractal Detrended Fluctuation Analysis (MFDFA) extends DFA to quantify heterogeneous scaling behaviors in signals whose local fluctuations cannot be described by a single Hurst exponent (Kantelhardt et al., 2002). MFDFA follows the same initial steps as DFA: the signal is first integrated, partitioned into segments of size s , and locally detrended to obtain the variances $F^{2}\left(\nu ,s\right)$ defined above.

To characterize fluctuations of different magnitudes, MFDFA introduces a moment order q∈R and defines the q -order fluctuation function as

$$F_{q}\left(s\right)={\left\{\frac{1}{2N_{s}}\overset{2N_{s}}{\underset{\nu =1}{\sum }}{\left[F^{2}\left(\nu ,s\right)\right]}^{q/2}\right\}}^{1/q},$$

with the case limq→0 is obtained using logarithmic averaging.

A multifractal signal exhibits the scaling law

$$F_{q}\left(s\right)∼s^{h\left(q\right)},$$

where h(q) is the *generalized Hurst exponent*. For monofractal signals, h(q) is constant; for multifractal signals, h(q) varies with q , reflecting different scaling behaviors for small versus large fluctuations. Hence, a dependence of h(q) on q indicates multifractality.

In this study, we applied MFDFA to 500 ms segments for each trial to capture the temporal evolution of multifractal properties. We used the generalized Hurst exponent as a feature in the classification framework. While full multifractal spectra (e.g., f(α) ) provide a more detailed characterization of nonlinear dynamics, including such high-dimensional descriptors would increase model complexity relative to the limited number of trials and may lead to overfitting. Therefore, the generalized Hurst exponent was used as a compact summary measure of multifractal behavior.

### 2.4 Fractional-order dynamical network models

A fractional-order dynamical network model (FODN) is a network whose behavior is described using differential equations of non-integer order, where the fractional derivative of a function at a given point depends on all its past values and thus naturally encodes long-range measurement dependencies (Reed et al., 2022). In this study, FODN is used to quantify memory dependencies in iEEG data, providing both temporal information through fractional-order dynamics and spatial information through the spatial matrix A , which describes how each channel influences the others and whose dominant eigenvector summarizes the network-level organization for each trial segment. For each channel $x_{i}\left[k\right]$ , a fractional-order exponent $\alpha_{i}$ is estimated using a Haar wavelet log-scale regression (Flandrin, 2002), where the slope $p_{i}$ determines $\alpha_{i}$ based on the following relationship

$$\alpha_{i}=\frac{p_{i}}{2}.$$

The discrete Grünwald–Letnikov fractional derivative (Chpt.1 (Akdemir et al., 2020)) is computed as

$$z_{i}\left[k\right]=\overset{J}{\underset{j=0}{\sum }}c_{j}\left(\alpha_{i}\right)x_{i}\left[k-j\right],$$

with coefficients

$$c_{j}\left(\alpha_{i}\right)=\frac{\Gamma \left(-\alpha_{i}+j\right)}{\Gamma \left(-\alpha_{i}\right)\;\Gamma \left(j+1\right)},$$

with Γ(⋅) denoting the Gamma function (Dzielinski and Sierociuk, 2005). Stacking across channels yields

$$z\left[k\right]=D^{\alpha }x\left[k\right],$$

which approximates the vector of fractional derivatives. The FODN model relates this fractional activity to the previous neural state through

$$z\left[k\right]=Ax\left[k-1\right]+Bu\left[k\right]+\varepsilon \left[k\right],$$

where A captures directed interactions among channels, B maps sparse perturbations, and u[k] represents transient inputs. An initial estimate of A is obtained by regularized least squares,

$$A=ZX^{⊤}{\left(XX^{⊤}+\lambda I\right)}^{-1},$$

and the sparse inputs are refined using the LASSO optimization

$$u\left[k\right]=\arg\underset{u}{\min}\left(\frac{1}{2}{\left\|z\left[k\right]-Ax\left[k-1\right]-Bu\right\|}_{2}^{2}+\lambda \|u{\|}_{1}\right).$$

By alternating between estimating u[k] and refitting A using the residual z[k]−Bu[k] , FODN yields a stable representation of memory-based neural interactions, enabling insight into how and where cognitive effort modulates epileptiform activity.

While the Hurst exponents computed using DFA provides a baseline characterization of long-range temporal correlations in individual iEEG time series, the fractional-order exponent (α) derived from the FODN model reflects temporal memory properties within a spatially coupled network framework. Although both measures relate to temporal scaling behavior, the Hurst exponets computed using DFA operates at the single-channel level, whereas FODN captures coupled spatial-temporal interactions across channels. MFDFA further complements these approaches by quantifying multifractal and nonlinear scaling behavior across multiple time scales. Together, these methods provide complementary perspectives on neural dynamics, with Hurst exponents computed using DFA serving as a reference measure for fractional-order exponents from FODN models, and MFDFA providing higher-dimensional features used for classification.

### 2.5 Dominant eigenvectors

For each trial segment, the FODN interaction matrix A is summarized using its dominant eigenvector. Let $A=E\Lambda E^{-1}$ , where E contains the eigenvectors and Λ the corresponding eigenvalues. The dominant eigenvector, associated with the eigenvalue of largest magnitude, represents the leading mode of the network dynamics. Electrodes with larger absolute weights in the dominant eigenvector exert greater influence on the overall network behavior.

Recent work suggests that dominant eigenvectors may point to the starting location of seizures in the brain (Roy et al., 2025). We hypothesize that changes in the spatial structure of eigenvectors across time and trial outcomes may reflect shifts in large-scale network organization induced by cognitive effort.

### 2.6 Trial-level classification using logistic regression

To evaluate whether the combined features extracted from FODN and multifractal analysis can reliably distinguish successful from failed trials, we developed a trial-level logistic regression classifier. Because the trial outcome is binary (successful vs. failed suppression of EAADs), logistic regression provides a linear framework for modeling the probability of each outcome. Given the relatively small number of trials available, a simple and interpretable model such as logistic regression helps reduce the risk of overfitting compared with more complex machine learning approaches.

For each trial, a feature vector was constructed using four key descriptors: the mean and variance (across time and all channels) of the FODN-derived fractional-order exponents (α) , the dominant eigenvector of the FODN coupling matrix, and the generalized Hurst exponent obtained from MFDFA. These features jointly capture temporal dependencies, spatial coupling, and multi-scale fluctuations of neural activity.

All features were normalized prior to model fitting, and classification performance was assessed using a leave-one-trial-out cross-validation strategy. At each fold (out of 43 folds), the model was trained on all remaining trials and tested on the held-out trial, providing an unbiased estimate of generalization under limited data conditions. Because the model was evaluated using leave-one-trial-out cross-validation on a limited number of trials, emphasis was placed on predictive generalization rather than detailed coefficient-level inference, as regression coefficients may vary across folds and may not provide stable physiological interpretation. This approach enables interpretable evaluation of how well the combined dynamical and multifractal features separate successful suppression of EAADs responses from failed ones.

## 3 Results

We demonstrate that the Hurst exponent captures temporal changes distinguishing successful from failed trials and that the dominant eigenvectors of FODN models provide spatial insight into how network organization evolves across time.

**FIGURE 2:**
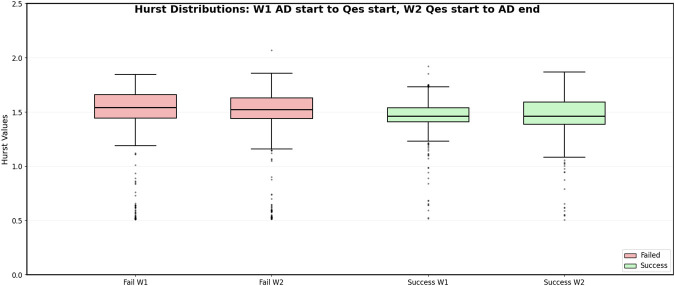
Hurst exponents of all channels computed with 500 ms non-overlapping time windows during W1 and W2 for both successful (green) and failed (red) trials.

Figure 2 shows the distribution of Hurst exponents computed across all channels during W1 and W2 for both successful and failed trials. Each data point represents the average across time Hurst exponent for a single channel within the corresponding window ( W1 or W2 ) of a given trial. For successful trials, pooling across all trials and channels yielded 1246 Hurst exponents in W1 and 1246 Hurst exponents in W2 . The Kolmogorov–Smirnov test comparing these two distributions resulted in a statistic of 0.10 and a p -value of $6.29\times 10^{-7}$ . For failed trials, the aggregated distributions contained 1795 Hurst exponents in W1 and 1795 Hurst exponents in W2 . The Kolmogorov–Smirnov test between W1 and W2 produced a statistic of 0.076 and a p -value of $6.65\times 10^{-5}$ for failed trials.

**FIGURE 3:**
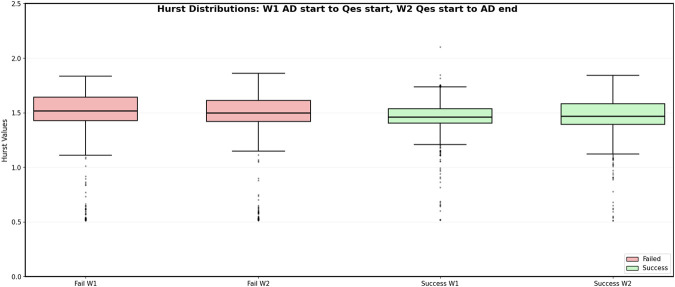
Hurst values of all channels computed with 500 ms 50% overlapping time windows during W1 and W2 for both successful (green) and failed (red) trials.

Figure 3 shows the distribution of Hurst values computed across all channels during W1 and W2 for both successful and failed trials using 50% overlapping windows. Each data point corresponds to an average across Hurst exponent for a single channel within the respective window ( W1 or W2 ) of a given trial. For successful trials, pooling across all channels and all trials yielded 1798 Hurst exponents for W1 and Hurst exponents 1798 for W2 . The Kolmogorov–Smirnov test comparing Hurst exponents in W1 and Hurst exponents in W2 resulted in a statistic of 0.10 and a p -value of $6.29\times 10^{-7}$ . For failed trials, the corresponding pooled distributions contained 2068 Hurst exponents in W1 and 2068 Hurst exponents in W2 . The Kolmogorov–Smirnov test between Hurst exponents in W1 and Hurst exponents in W2 yielded a statistic of 0.076 and a p -value of $6.65\times 10^{-5}$ for failed trials.

**FIGURE 4:**
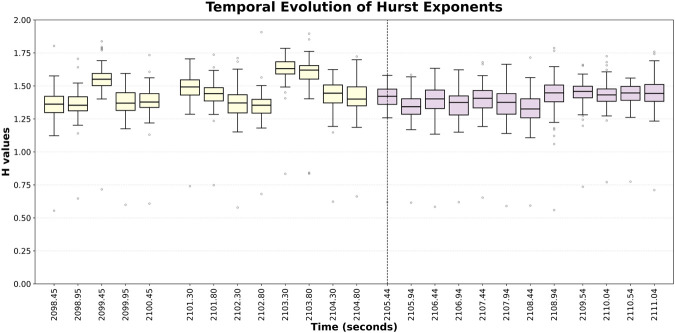
Hurst exponents across 500 ms chunks for Patient 2, Trial 7 a successful trial.

Figure 4 illustrates the temporal evolution of Hurst exponents computed over 500 ms chunks for Patient 2, Trial 7, a successful trial. We observe that the Hurst exponents fluctuate widely during W1 before the W2 phase, showing transient irregularity during EAADs before cognitive effort is exerted. Once W2 begins, the period from question start to after discharge end, the variance of the Hurst exponents diminish, indicating that the iEEG signals become more consistent during cognitive effort exertion and post-discharge. While Figure 4 shows a single trial from a single patient, we observe this pattern of the Hurst exponents to be a consistent trend across successful trials (see Supplementary Material), although additional data across more trials may be needed to draw firm conclusions.

Since the analysis is performed using 500 ms non-overlapping windows, the appearance of intermediate event markers (e.g., Qes_End, Ans_Start, Ans_End) causes the windowing to restart at those time points. This produces small discontinuities in the computation of Hurst exponents and eigenvectors across time, which reflect issues with the experimental timing rather than any issue with the analysis itself.

**FIGURE 5:**
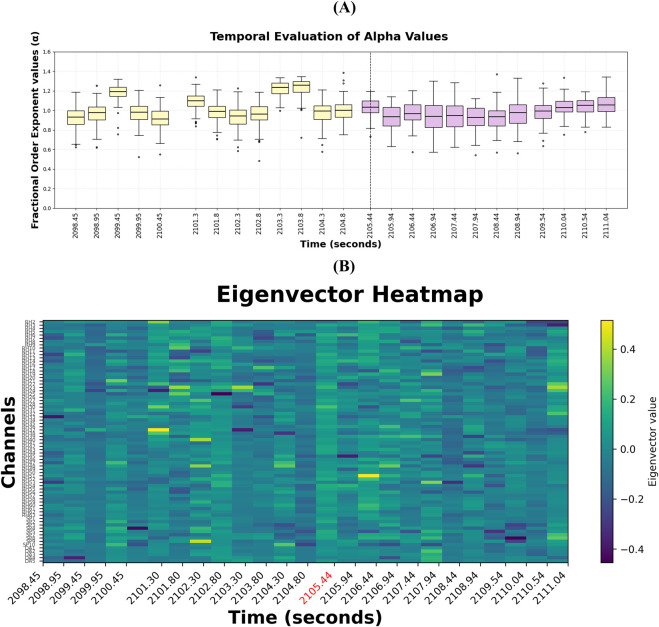
**(A)** Fractional-order exponents (α) distributions across 500 ms chunks for Patient 2, Trial 7. **(B)** FODN-based dominant eigenvector heatmap for Patient 2, Trial 7. Rows correspond to electrode channels and columns correspond to time windows, while the color scale represents each channel’s contribution to the dominant eigenvector, illustrating how the spatial influence of channels evolves over time.

Figure 5a shows the temporal evolution of the fractional-order exponents (α) for Patient 2, Trial 7. During the W2 phase, the fractional-order exponents (α) become more consistent across time, reflecting a stabilization of temporal scaling behavior after cognitive effort is exerted and suppression of EAADs is achieved. This temporal regularity observed in fractional-order exponents aligns with the emergence of more controlled neural dynamics during the exertion of cognitive effort and suppression period observed in Figure 1. Notably, the temporal evolution of the Hurst exponent exhibits the same pattern during W2 as the fractional-order exponents, providing independent validation that neural dynamics during cognitive effort and suppression of EAADs become more orderly and regulated.

Figure 5b presents the dominant eigenvector heatmap, illustrating the temporal evolution of the spatial contributions of individual electrode channels to the dominant dynamical mode in the fractional-order dynamical network model. Each column corresponds to the dominant eigenvector estimated for a single time window, and each row represents an electrode channel. The color scale reflects each channel’s contribution to this mode, offering a compact view of how spatial influence is distributed across the network. Channels with strongly positive or negative values (bright yellow or deep purple, respectively) emerge as dominant contributors, capturing shifts in which channels act as network hubs, periods of transient recruitment, and broader changes in network organization.

Several spatial features are apparent. First, most channels remain clustered near small values (green), consistent with networks in which only a subset of nodes drive the global dynamics. Second, occasional horizontal bursts reflect brief periods where individual channels momentarily dominate the network, indicating transient spatial reconfigurations. Finally, the spatial structure reflects physiologically meaningful neural coupling between iEEG channels rather than artifacts, enabling clearer interpretation of network-level interactions.

**FIGURE 6:**
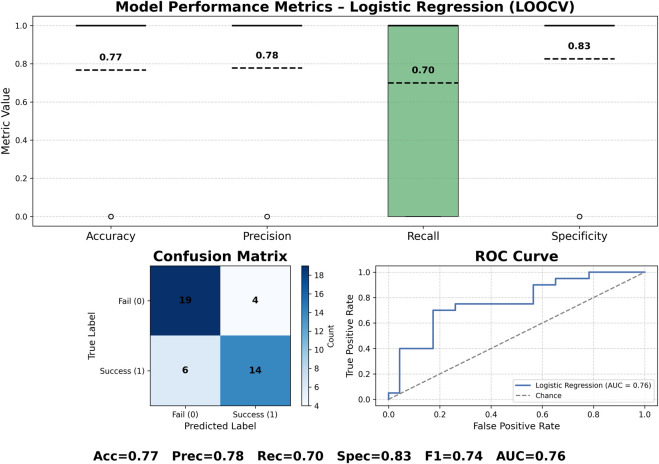
Logistic regression performance using LOOCV, confusion matrix, per-fold performance metrics, and ROC curve (AUC = 0.76).

Figure 6 summarizes the performance of the logistic-regression model trained on features derived from FODN and multifractal analysis. Our logistic regression model achieves an overall accuracy of 0.77, correctly predicting 33 of 43 trials under leave-one-out cross-validation, demonstrating that these spatial-temporal features carry meaningful discriminative power. Our results show consistent precision, recall, and accuracy across folds, as well as reliable detection of both failure and success outcomes via model performance metrics and confusion matrix. Notably, achieving this level of performance with a relatively small number of available trials suggests that the chosen features are robust indicators of trial outcome and hold promise for future predictive modeling as more data become available.

## 4 Discussion

### 4.1 Spatial-temporal framework captures important features to determine successful and failed trials

Our work characterizes how cognitive effort influences both temporal and spatial properties of neural dynamics during suppression of EAADs. By combining Hurst exponents, generalized Hurst exponents, and FODN-based eigenvectors, we provide a unified framework to capture multi-scale and network changes in brain activity leading up to and during cognitive effort and the suppression of EAADs. The Hurst exponents computed using DFA and generalized Hurst exponents computed using MFDFA results indicate changes in long-range temporal dependencies and multi-scale variability, while the eigenvectors from FODN models captures how these dynamics are organized across spatially distributed neural networks.

Based on the results in Figures 2, 3, detecting changes between W1 and W2 for successful and failed trials is difficult when the Hurst exponents are averaged across time. In fact, for successful trials, changes in Hurst exponents over time are more noticeable before cognitive effort is exerted, where the mean and variance of the Hurst exponents fluctuate greatly across channels, as seen in Figure 4. After cognitive effort is exerted, the Hurst exponents are more consistent in mean and variance (see Figure 4). These observations suggest that the temporal changes before and during cognitive effort are important to analyze and cannot be effectively summarized across time. Furthermore, the results suggest that the fractal properties of the brain activity varies widely before cognitive effort is exerted, indicating that cognitive effort may promote less chaotic behavior leading to effective suppression of EAADs. Figure 5a supports this notion since the fractional-order exponents reflect a similar trend shown by the Hurst exponents. In most patients and trials, the fractional-order exponents and Hurst exponents exhibit higher variance and means during W1 as compared with W2 in successful trials (see Supplementary Material). Finally, although we did not explicitly quantify statistical associations between temporal scaling metrics and network-level features, our results consistently show that successful suppression trials exhibit both reduced variability in Hurst and fractional-order exponents and increased consistency in network-level representations (e.g., dominant eigenvectors). This co-occurrence suggests that temporal variation and network-level organization are closely linked aspects of the same underlying neural process.

Our work demonstrates that our framework, which computes spatial-temporal features using DFA, MFDFA, and FODN models, carry meaningful discriminative power to predict successful versus failed trials. Figure 6 shows the performance of our logistic regression model, which achieves an average accuracy of 0.77, where it correctly predicted 33 of 43 trials under leave-one-out cross-validation. Our results show high precision, recall, and accuracy across folds. Furthermore, our results demonstrate reliable detection of both failure and success outcomes, reporting low false positive and false negative results shown in the confusion matrix in Figure 6. Our framework produces robust performance despite having a relatively small number of available trials to train and test. Hence, our framework holds promise for future predictive modeling as more data become available.

### 4.2 Suppression of EAADs during cognitive effort likely due to a coordinated network effect

Recent work has suggested that seizure suppression potentially involves more than one brain region and in fact may be a network effect (Bassett and Sporns, 2017; Ching et al., 2012). However, cognitive tasks are believed to have specific brain regions associated with their execution. For example, a study aiming to map regions of the brain responsible for language suggested that a broad network may be responsible (Matsumoto et al., 2004). Another study showed that reading and spelling tasks share specific left hemisphere substrates in the mid-fusiform gyrus and in the inferior frontal gyrus/junction, with lexical orthographic processing occurring in the left mid-fusiform substrates (Rapp and Lipka, 2011). Additionally, the study in (Dehaene et al., 2005) suggested that the intraparietal sulcus is activated whenever numbers are manipulated, and the left angular gyrus supports the manipulation of numbers in verbal form. However, no study to our knowledge adequately quantifies the relationship between activity in these and other circuits with their ability to suppress epileptic activity.

Our results suggest that suppression of EAADs during cognitive effort is likely due to a coordinated network effect as opposed to an isolated event or particular brain region. These findings are consistent with principles of Network Physiology (Ivanov, 2021; Bashan et al., 2012), where physiological function arises from interactions across systems and scales rather than isolated subsystems. In this context, successful suppression of EAADs reflects coordinated changes in temporal dynamics and network organization, suggesting that cognitive effort globally modulates brain network dynamics. The observed changes in Hurst exponents, fractional-order exponents (α) , and dominant eigenvectors indicate widespread modulation of temporal dynamics and network coupling rather than purely local effects. The temporal evolution of fractional-order exponents (α) and Hurst exponents indicates that cognitive effort and suppressed EAADs dynamics become more consistent in mean and variance, supporting the hypothesis that cognitive effort promotes neural regulation. However, larger datasets with detailed spatial annotations will be necessary to validate the robustness of these network-level effects and to more confidently link them to underlying neural mechanisms.

These findings highlight that successful suppression is associated with coordinated changes across both temporal scaling behavior and network-level interactions, rather than isolated local effects. Importantly, the combination of temporal and spatial features provides a more comprehensive understanding of how cognitive effort modulates epileptic activity.

From a broader perspective, these results suggest the potential for developing non-invasive, cognitively driven approaches for modulating epileptic activity. Furthermore, the ability to quantify these changes using interpretable features may support the development of predictive models or real-time monitoring systems for identifying and enhancing suppression mechanisms.

### 4.3 Limitations

Due to the limited number of successful trials available for analysis, we are unable to reliably associate any channel-wise patterns observed from dominant eigenvectors with specific brain regions. Consequently, we could not determine which brain regions are most directly involved in the observed suppression of EAADs. Additional successful trials with well-annotated anatomical channel information will be essential to identify the brain regions that contribute to this suppression.

We note that the Hurst exponents computed using DFA reported in this study are larger than those typically reported in other EEG studies, where values are often in the range of 0.7–1 (Linkenkaer-Hansen et al., 2001; Hardstone et al., 2012). Our analysis yields Hurst exponents that in some cases approach 1.5 or higher. This difference likely arises from the nature of the analyzed signals, which consist of short, transient intracranial EEG segments during stimulation-induced electrical-discharges. These signals are inherently nonstationary and may exhibit integrated or strongly persistent dynamics, leading to higher scaling exponents. Similar behavior has been reported in physiological signals and in studies examining the effects of nonstationarity and signal preprocessing on power-law scaling properties (Hu et al., 2001; Chen et al., 2005; Ivanov et al., 1999). Therefore, the elevated Hurst exponents observed here should be interpreted as reflecting transient, large-scale temporal organization during EAADs rather than classical scale-free oscillatory activity.

## 5 Conclusion and future work

This study demonstrates that the fractional-order exponents (α) , the dominant eigenvectors, and the Hurst exponents each exhibit systematic changes during pre- and post-questioning. Together, these temporal ( α , Hurst) and spatial (eigenvectors) measures indicate that the dynamics of cognitive effort are quantifiable and sensitive to EAAD effects.

The performance results of our logistic regression model (see Figure 6) show that our framework identifies important multi-fractal and spatial-temporal features of the data to effectively classify successful and failed trials. Despite the limited number of available trials, the model achieved an accuracy of 0.77 under leave-one-out cross-validation with no evidence of extreme bias or overfitting. This performance suggests that the combined temporal-spatial features derived from FODN and MFDFA encode meaningful information about the suppression of EAADs during cognitive effort. In particular, the findings support the view that cognitive processing and suppression of EAADs are shaped by distributed network mechanisms rather than purely focal neural activity, consistent with principles of Network Physiology.

Future work will focus on identifying when and where cognitive effort most strongly impacts EAADs and determining the features most strongly associated with this effect. Doing so can also allow for a better understanding of the underlying physiological mechanisms of cognitive effort and its relationship to EAAD suppression.

This paper presents evidence for using cognitive effort as a new method for seizure suppression that minimizes cost, invasive procedures, and inconvenience for epileptic patients.

## Data Availability

The datasets generated and analyzed for this study are available from the corresponding author upon reasonable request and subject to institutional and ethical approvals.
