# Supplementary material for: Quantifying cognitive effort’s impact on suppression of epilepsy-associated after discharges
Source: Front Netw Physiol. 2026 May 19;6:1768476. doi: 10.3389/fnetp.2026.1768476 (PMC13226027; doi:10.3389/fnetp.2026.1768476)
Supplement: Supplementary file 1 [file DataSheet1.pdf]

Supplementary Material

1 SUPPLEMENTARY FIGURES

This file contains all supplementary figures referenced in the main manuscript, organised into three groups:

1. Eigenvector heat maps for successful trials.
2. Temporal evolution of fractional-order exponent α (successful trials)
3. Temporal evolution of Hurst exponents for successful trials.

1.1 Eigenvector heat maps (successful trials)

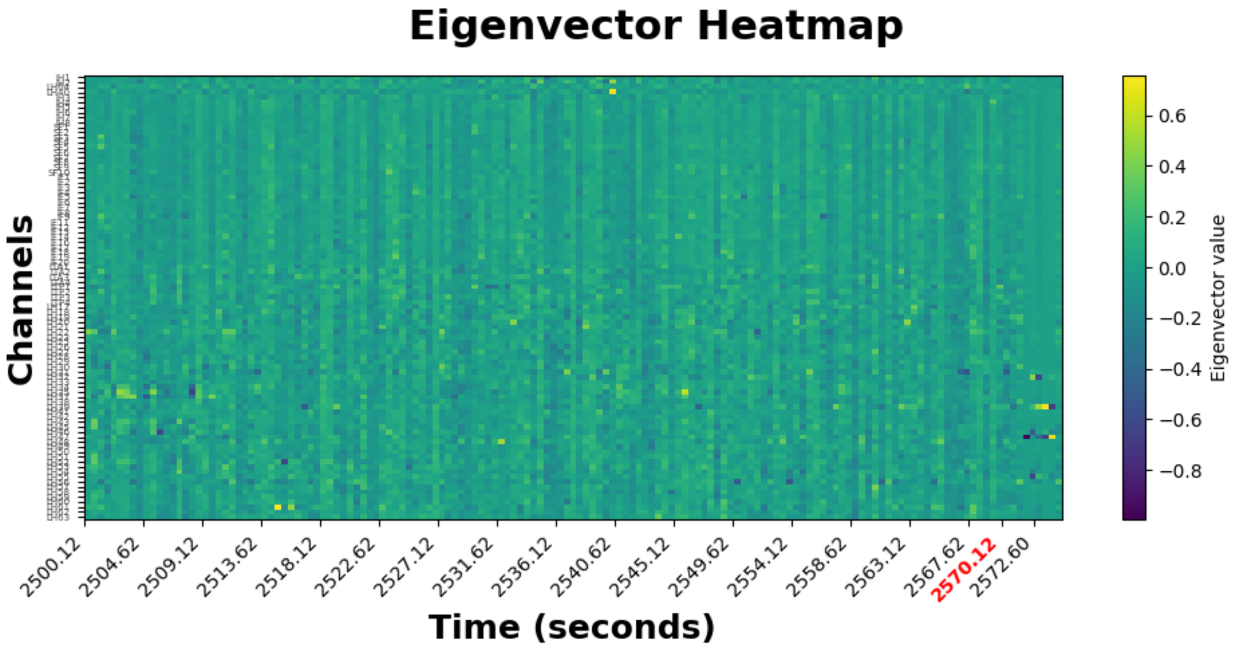

Figure S1. Eigenvector heat map for Patient 1, Session 5, Trial 1 (successful trial).

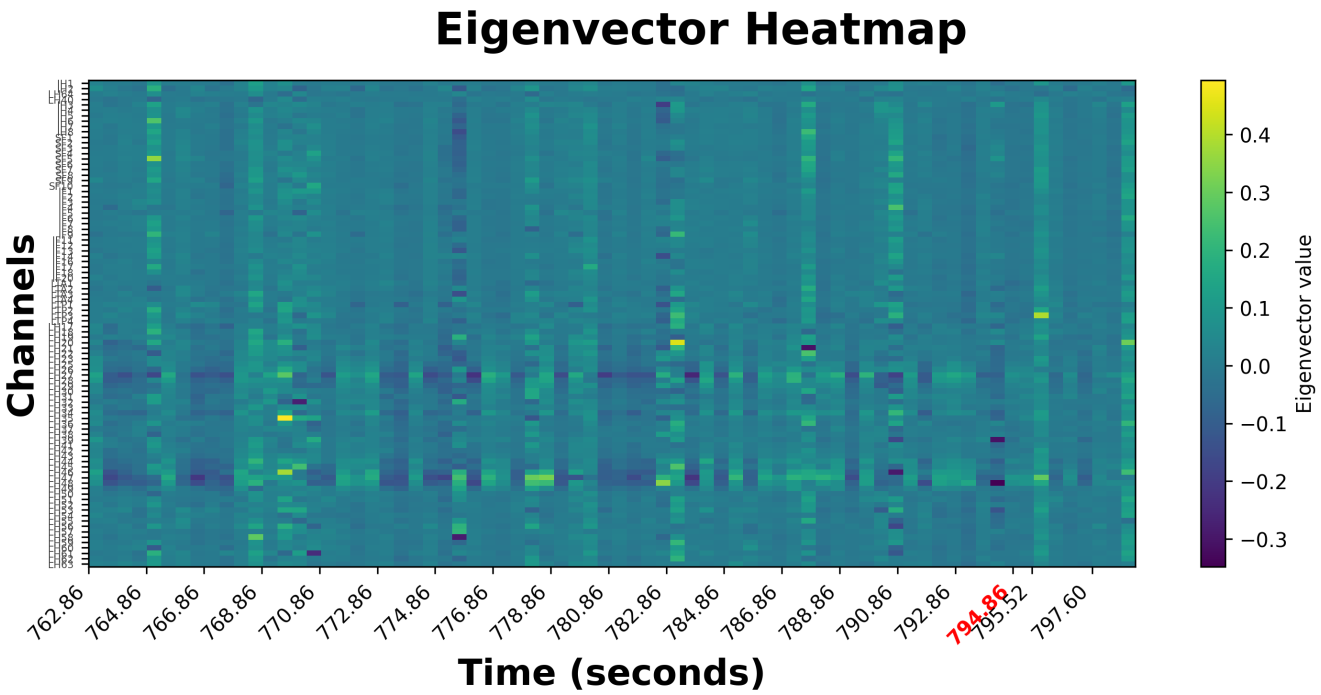

Figure S2. Eigenvector heat map for Patient 1, Session 6a, Trial 4 (successful trial).

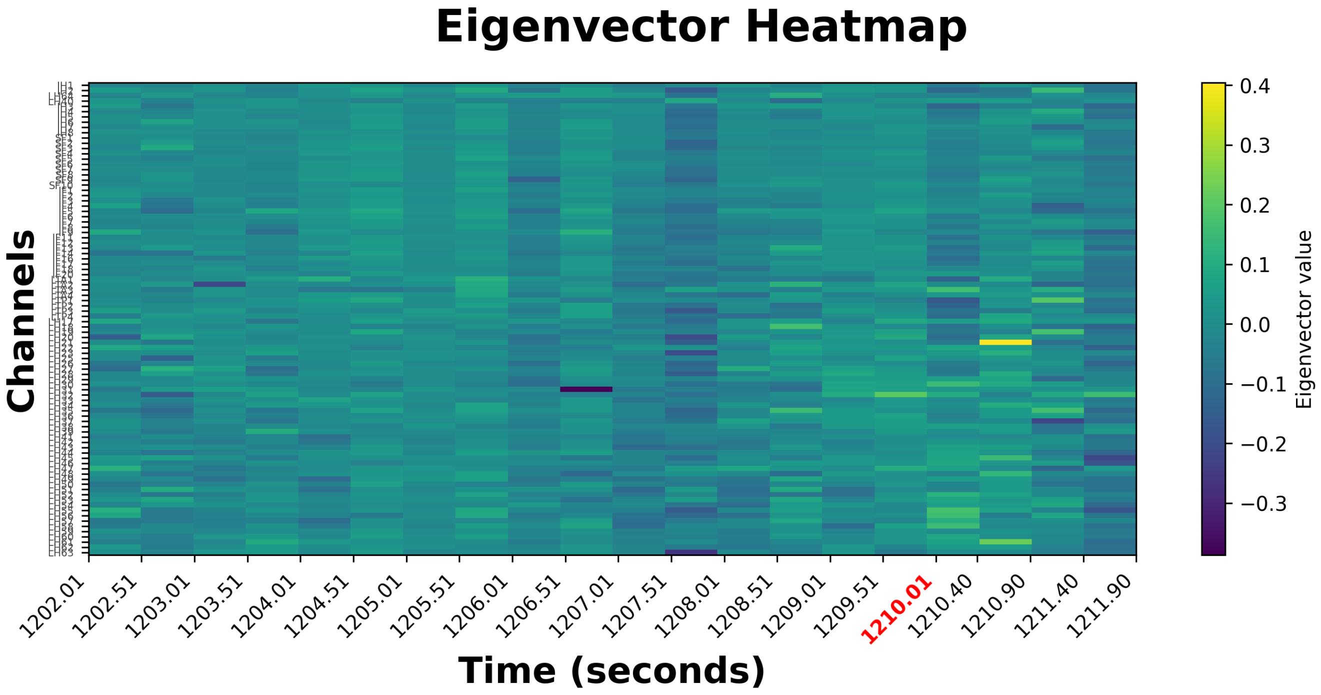

Figure S3. Eigenvector heat map for Patient 1, Session 6a, Trial 5 (successful trial).

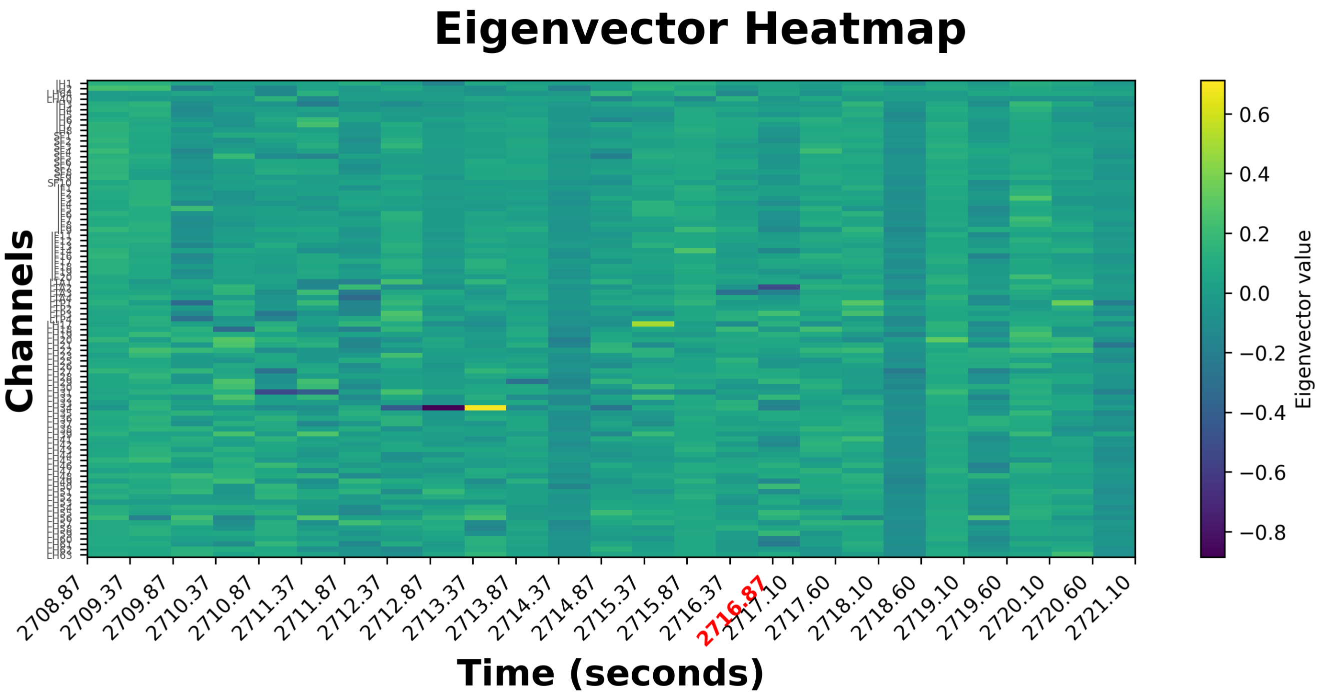

Figure S4. Eigenvector heat map for Patient 1, Session 6b, Trial 8 (successful trial).

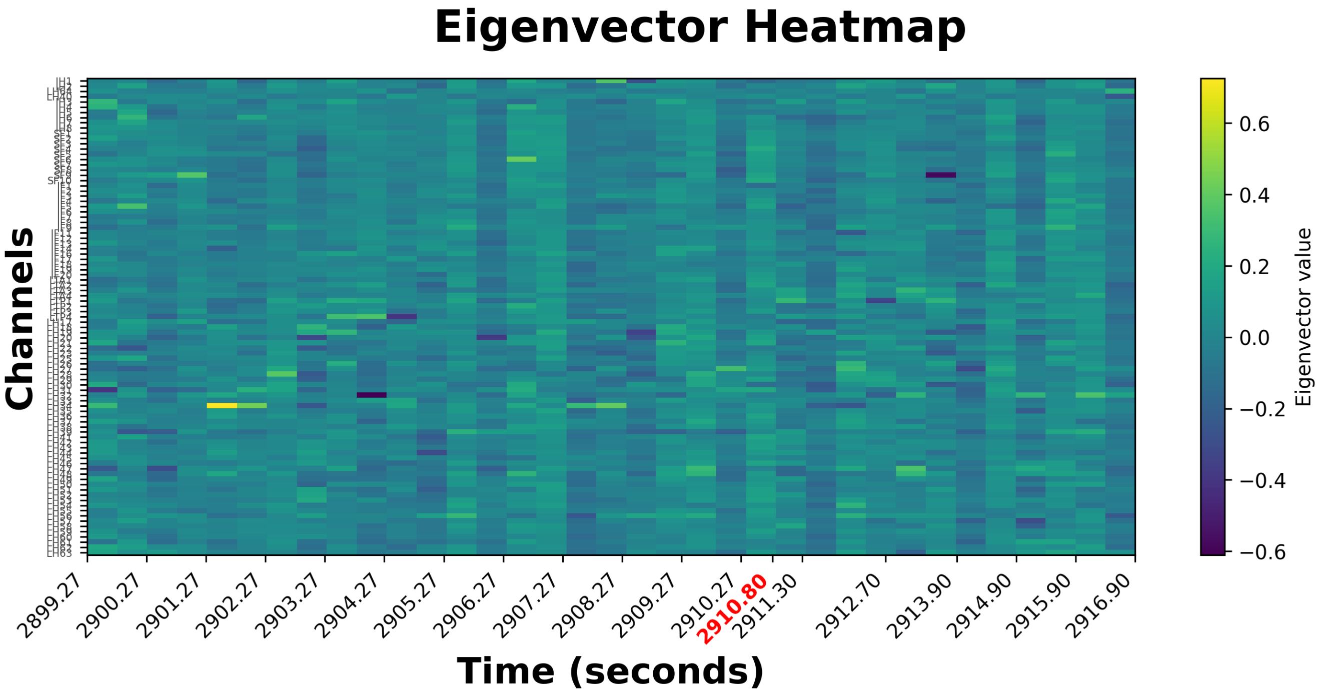

Figure S5. Eigenvector heat map for Patient 1, Session 6b, Trial 12 (successful trial).

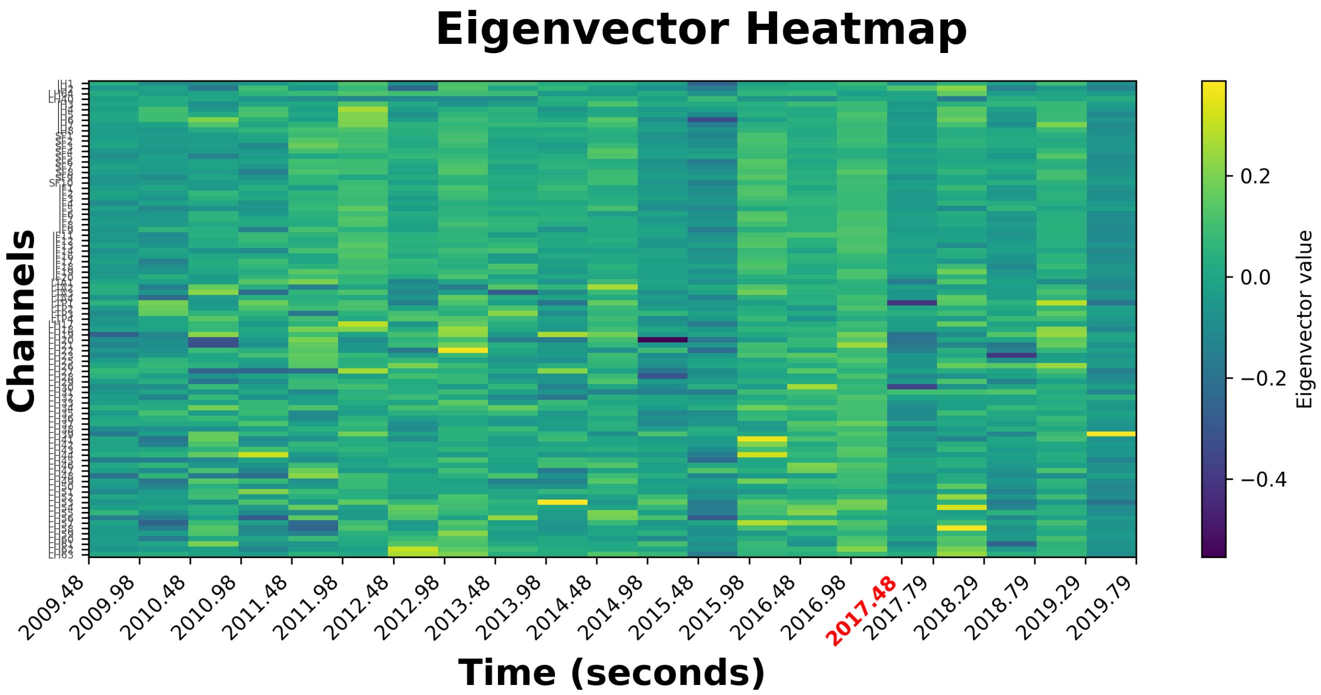

Figure S6. Eigenvector heat map for Patient 1, Session 8, Trial 28 (successful trial).

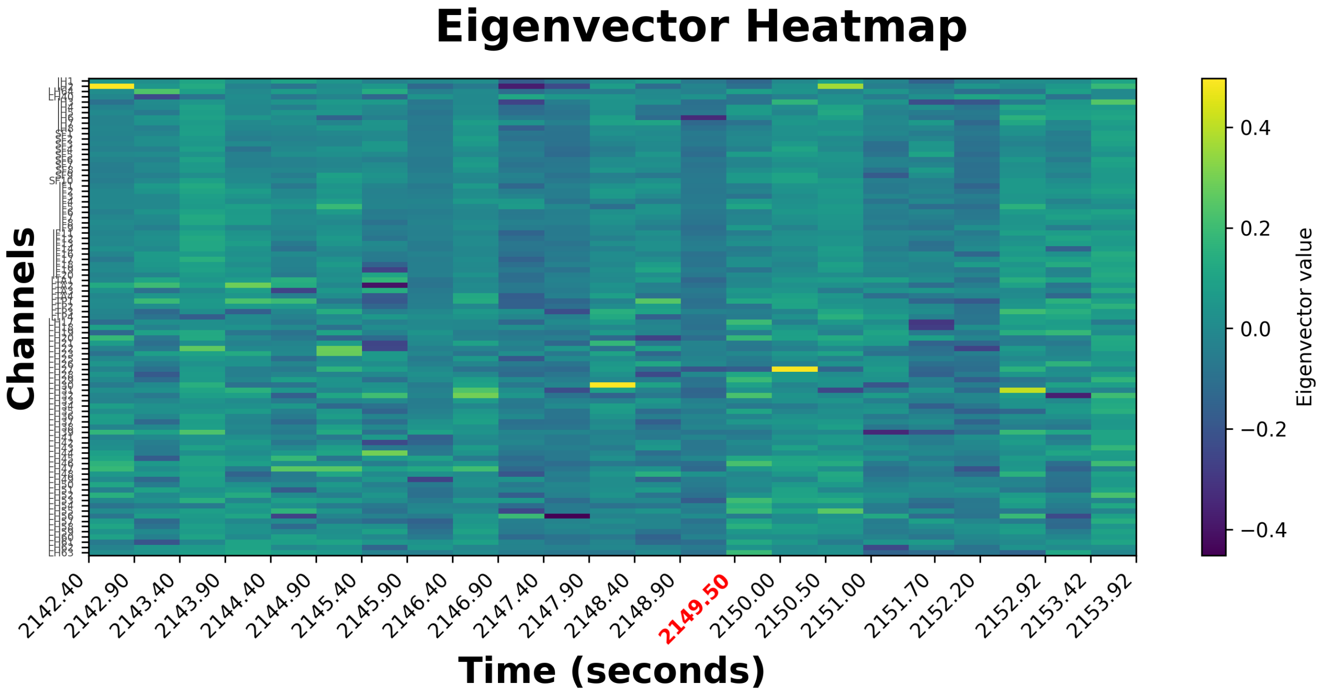

Figure S7. Eigenvector heat map for Patient 1, Session 8, Trial 31 (successful trial).

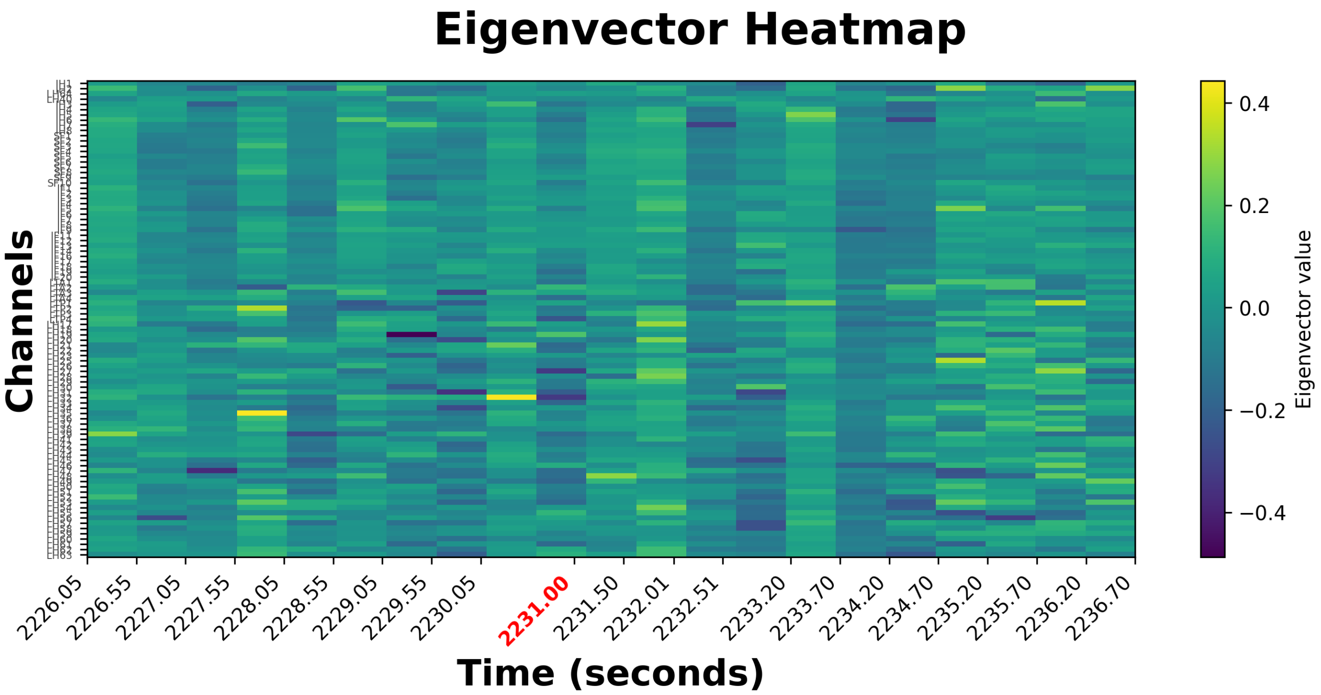

Figure S8. Eigenvector heat map for Patient 1, Session 8, Trial 32 (successful trial).

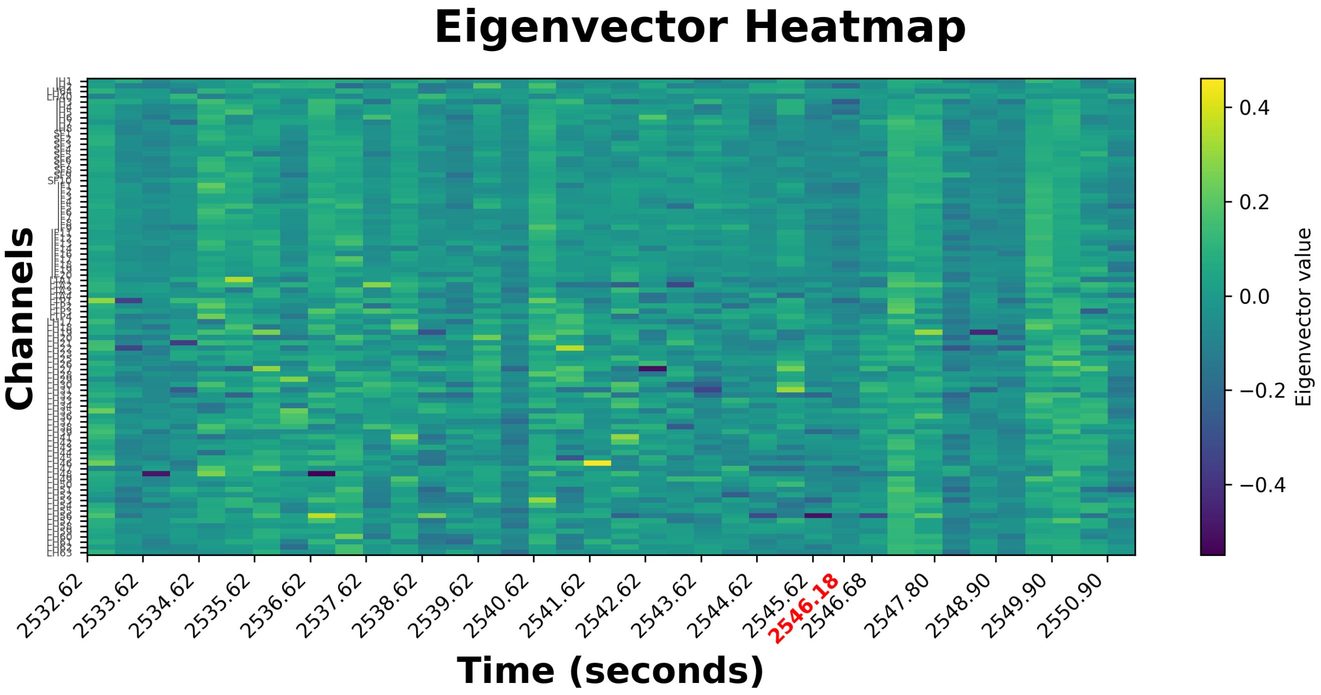

Figure S9. Eigenvector heat map for Patient 1, Session 8, Trial 37 (successful trial).

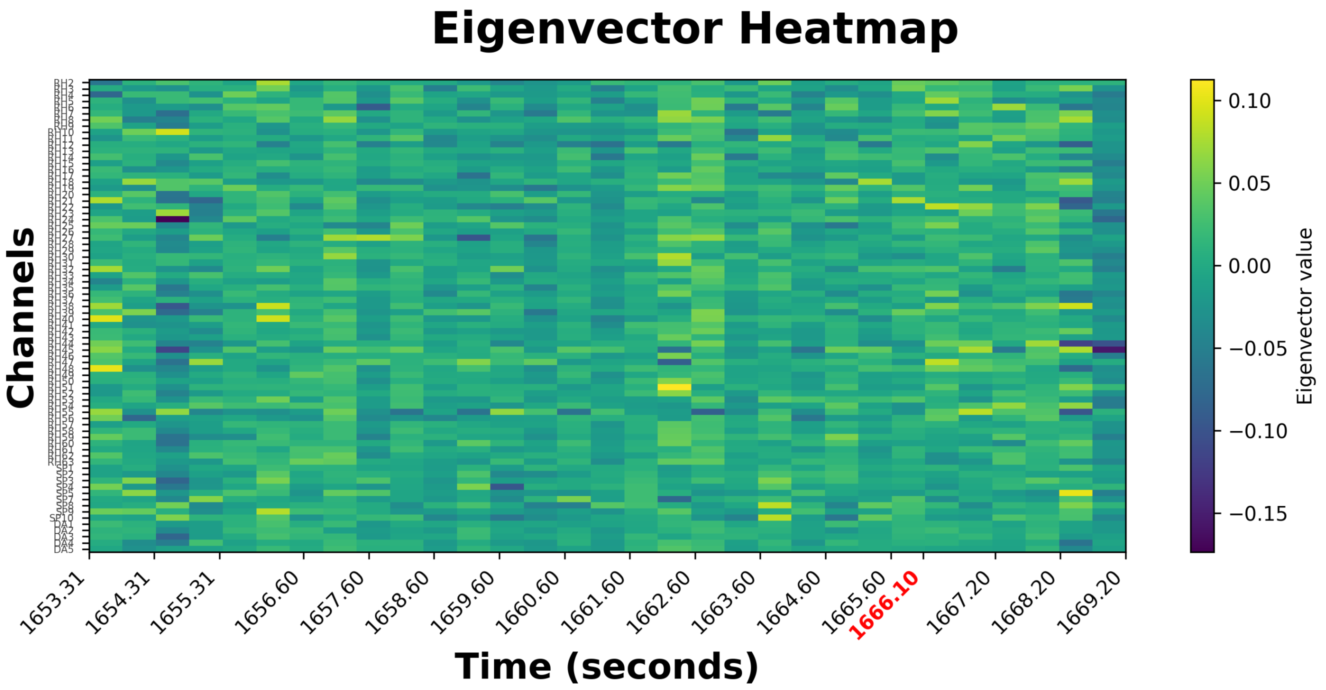

Figure S10. Eigenvector heat map for Patient 2, Session 3, Trial 4 (successful trial).

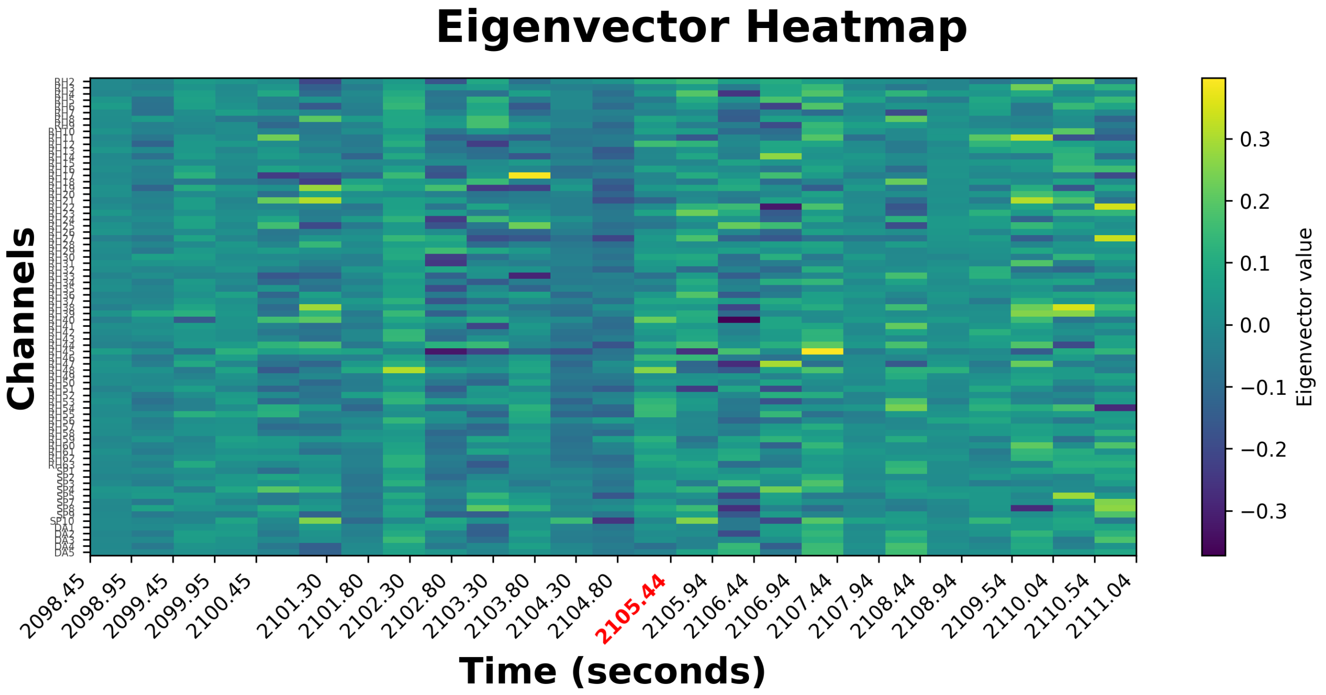

Figure S11. Eigenvector heat map for Patient 2, Session 3, Trial 7 (successful trial).

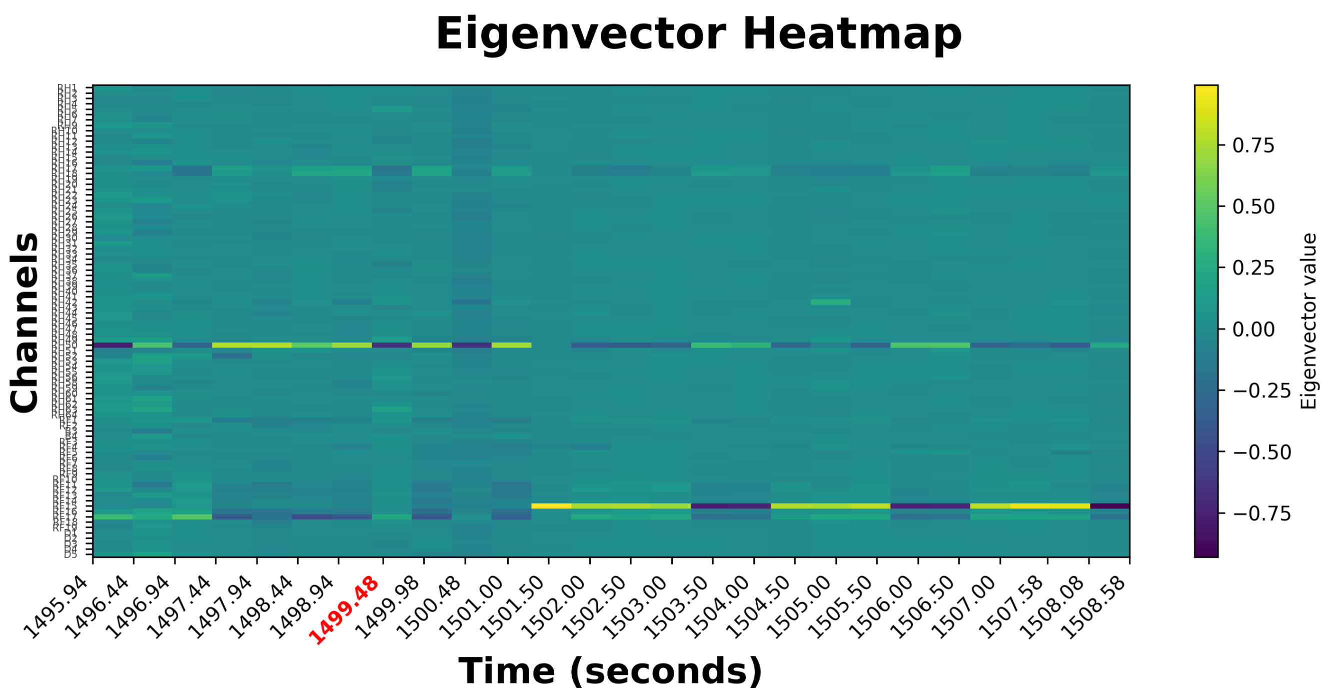

Figure S12. Eigenvector heat map for Patient 3, Session 2, Trial 5 (successful trial).

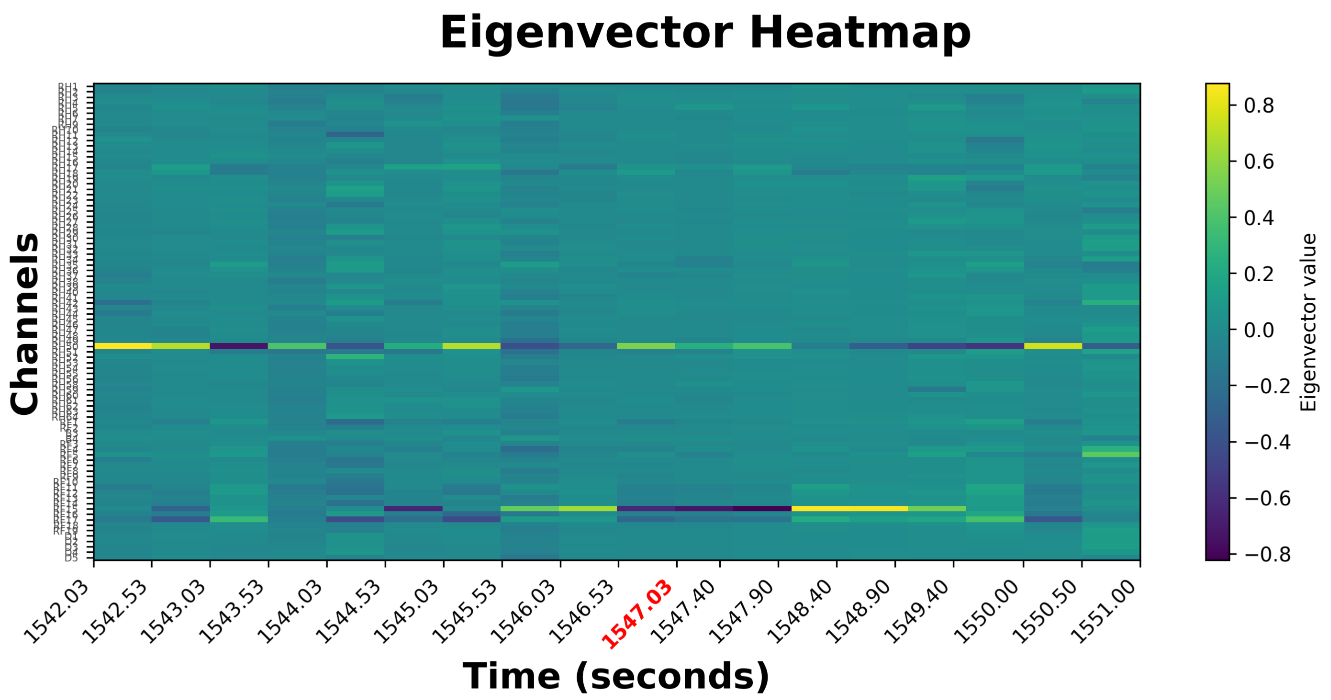

Figure S13. Eigenvector heat map for Patient 3, Session 2, Trial 6 (successful trial).

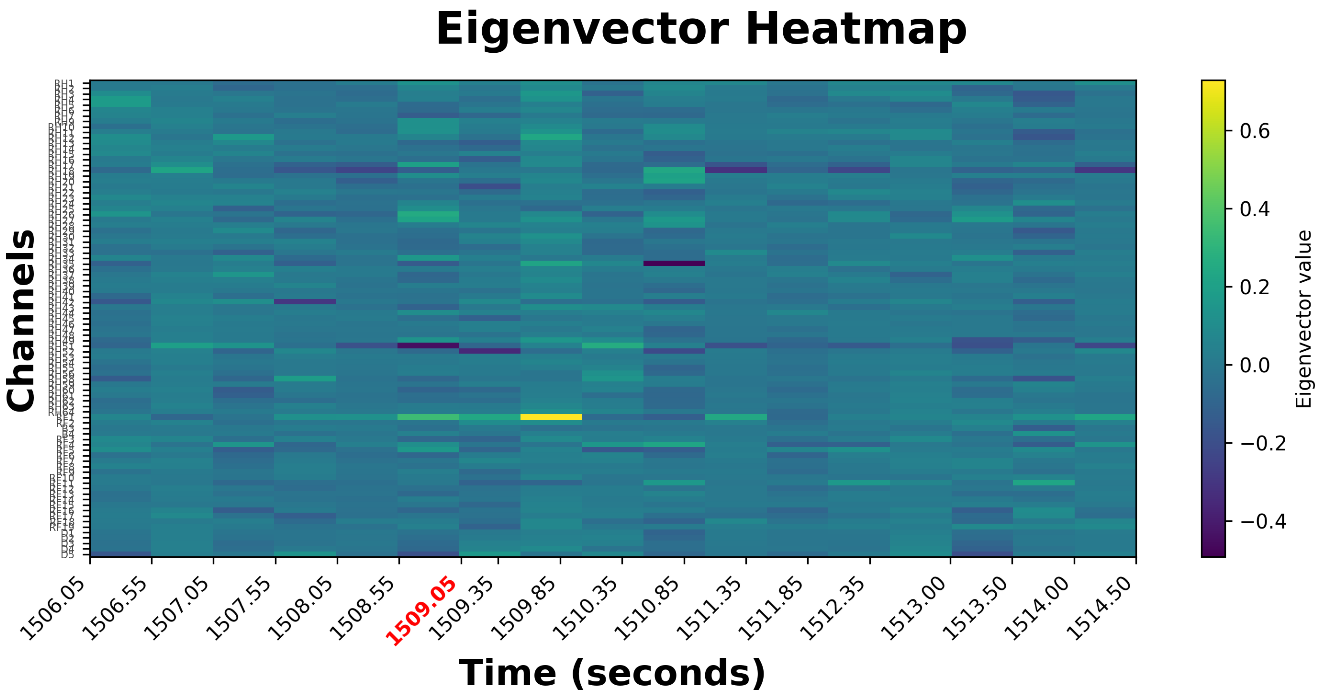

Figure S14. Eigenvector heat map for Patient 3, Session 3, Trial 8 (successful trial).

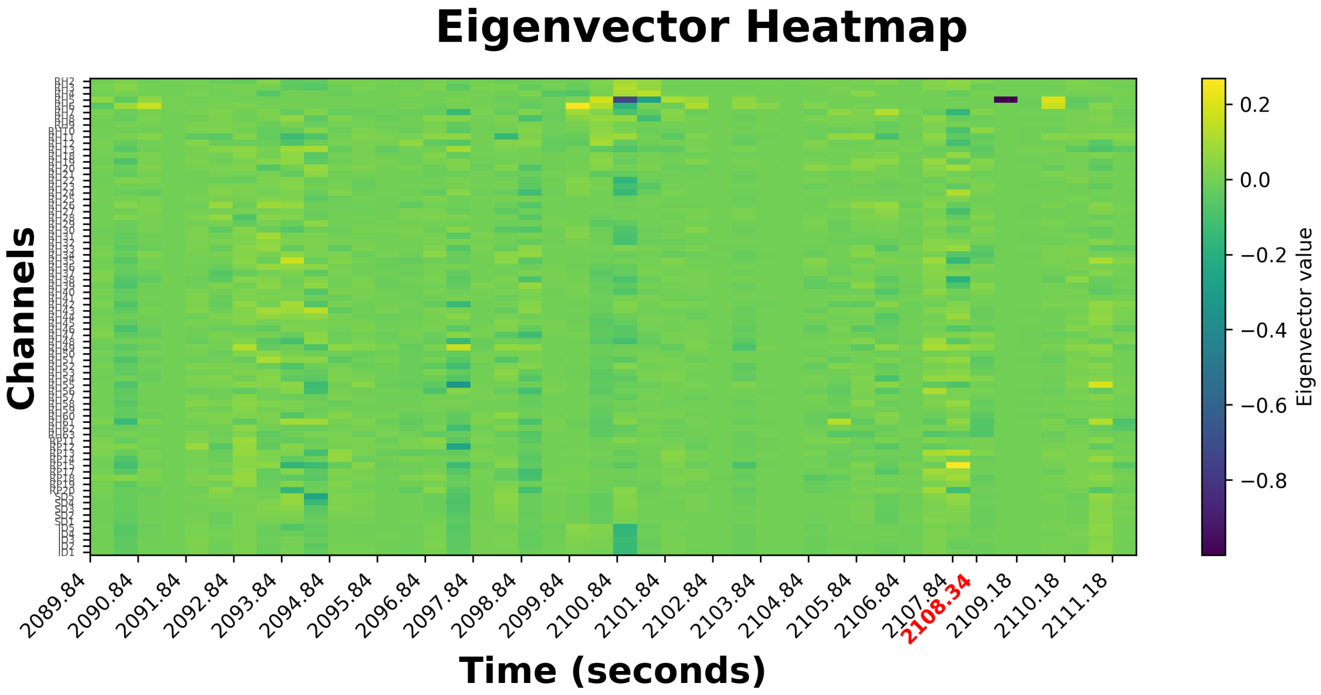

Figure S15. Eigenvector heat map for Patient 5, Session 1, Trial 1 (successful trial).

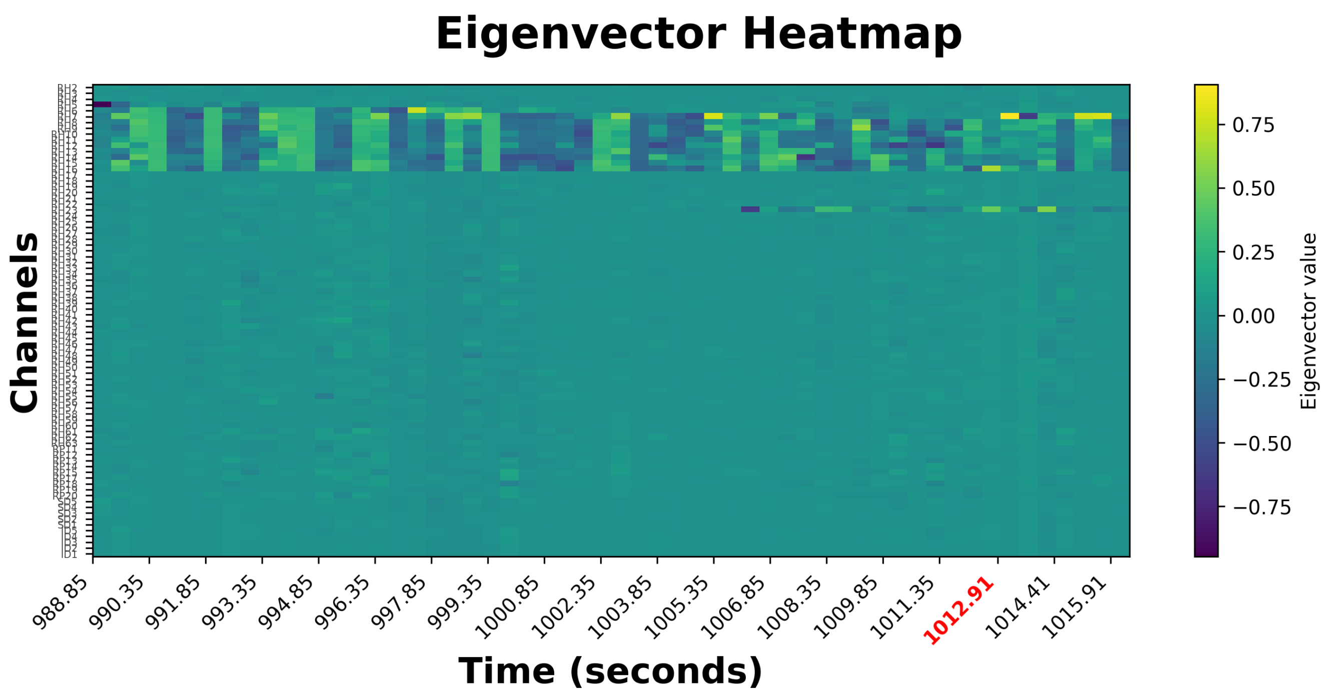

Figure S16. Eigenvector heat map for Patient 5, Session 2, Trial 2 (successful trial).

1.2 Temporal evolution of Hurst exponents (successful trials)

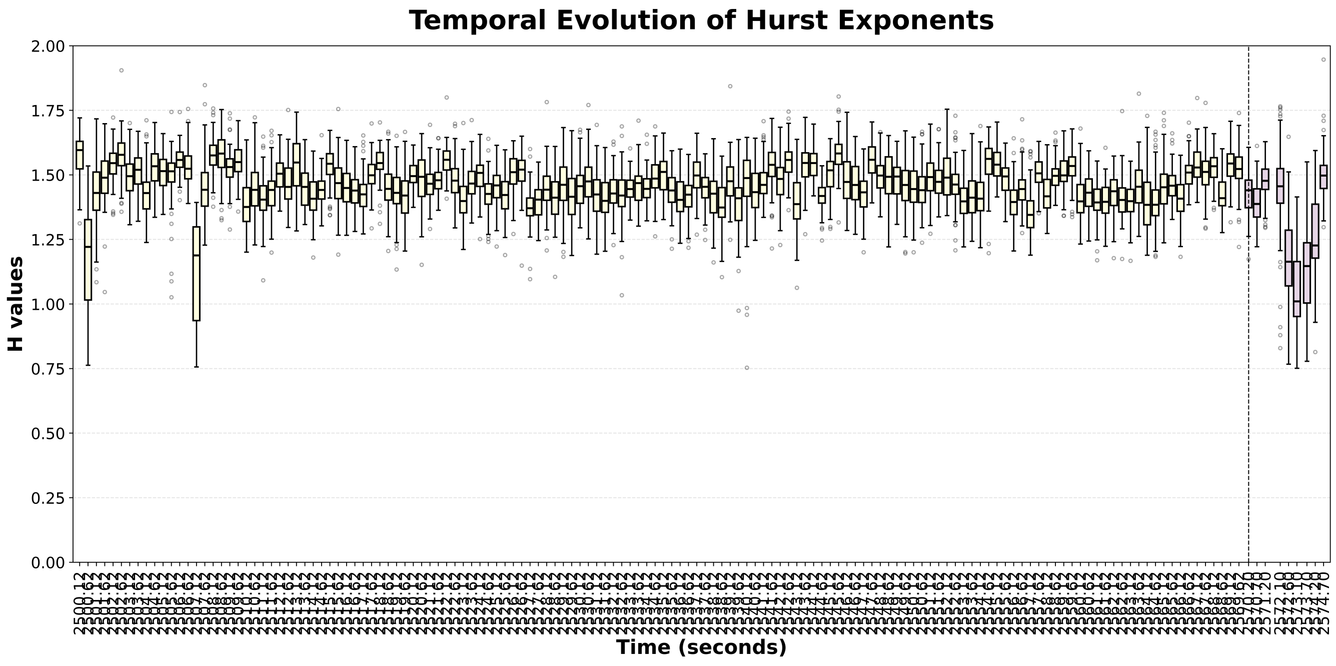

Figure S17. Temporal evolution of Hurst exponents for Patient 1, Session 5, Trial 1 (successful trial).

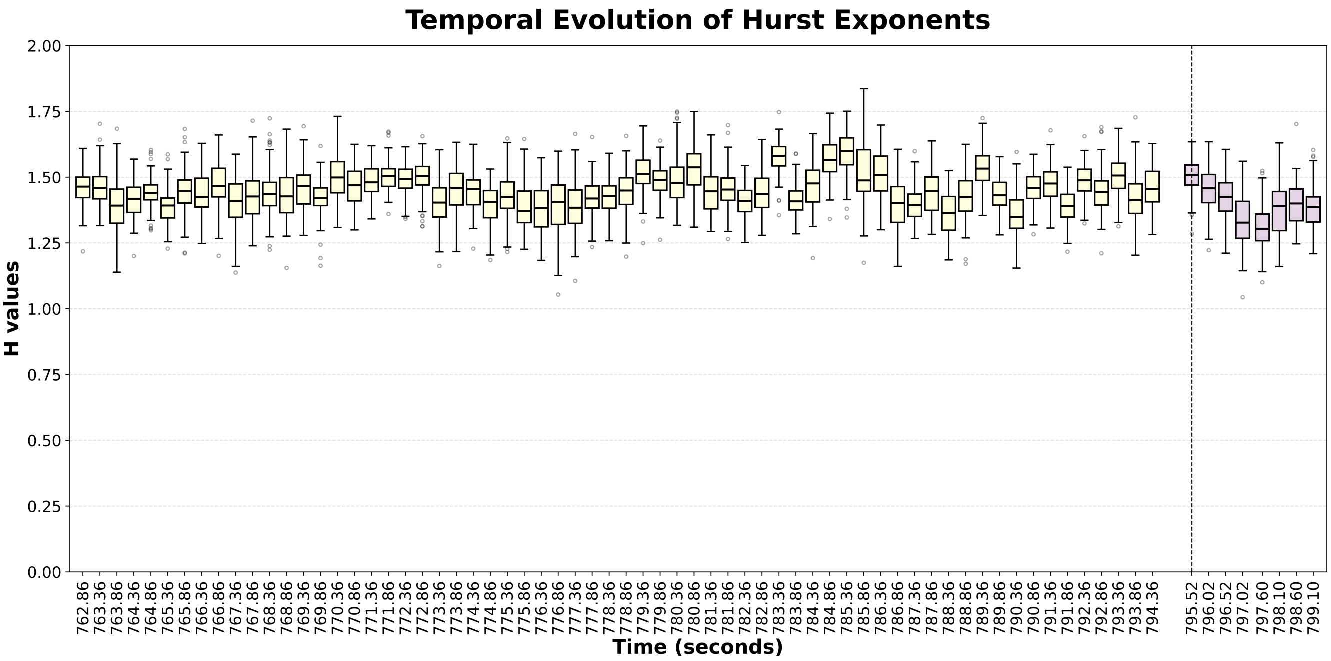

Figure S18. Temporal evolution of Hurst exponents for Patient 1, Session 6a, Trial 4 (successful trial).

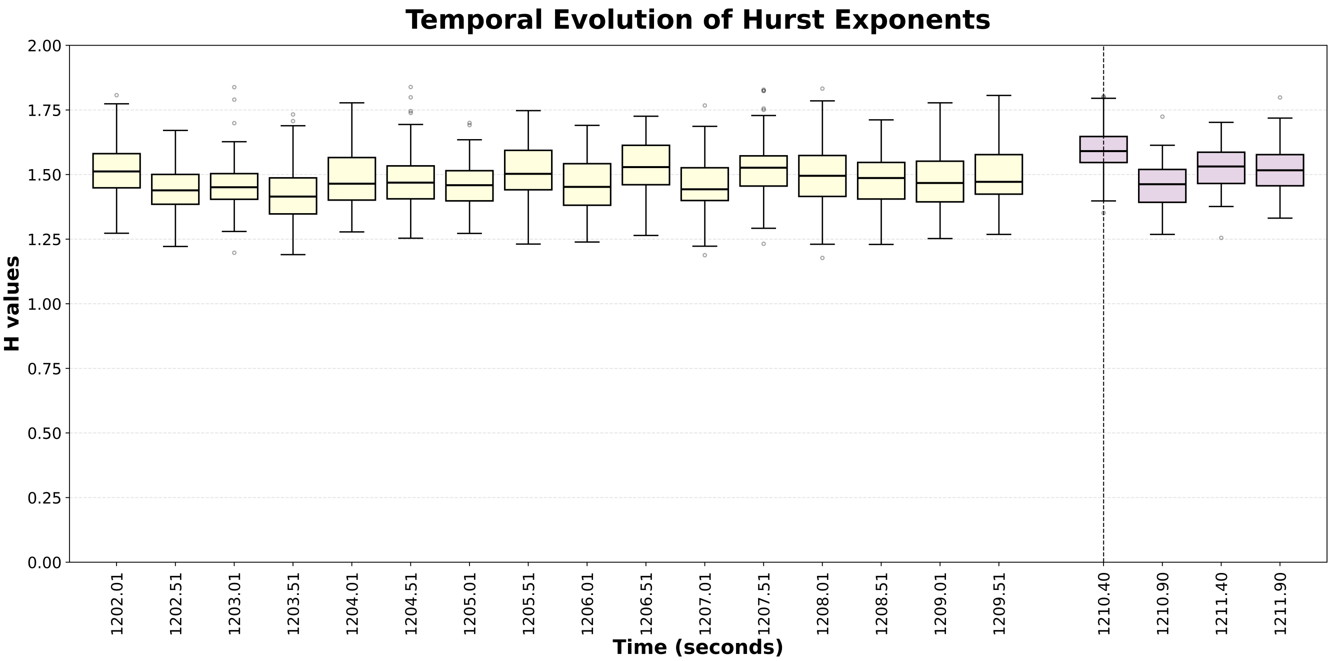

Figure S19. Temporal evolution of Hurst exponents for Patient 1, Session 6a, Trial 5 (successful trial).

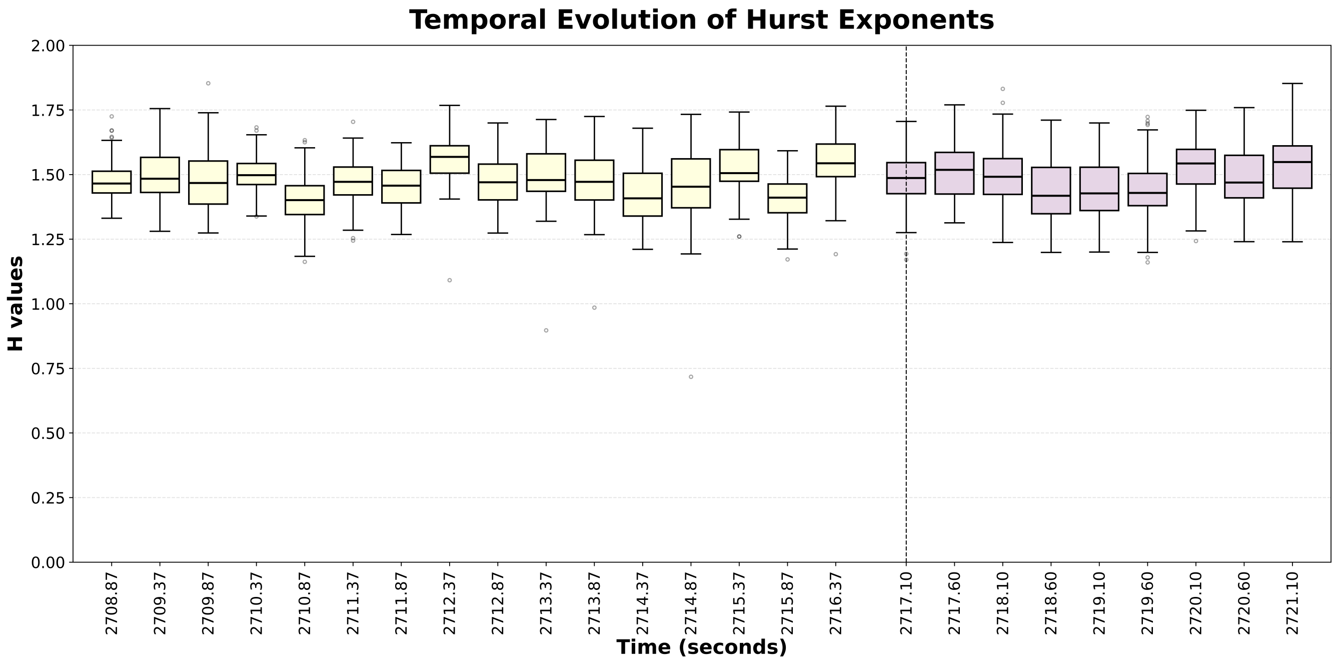

Figure S20. Temporal evolution of Hurst exponents for Patient 1, Session 6b, Trial 8 (successful trial).

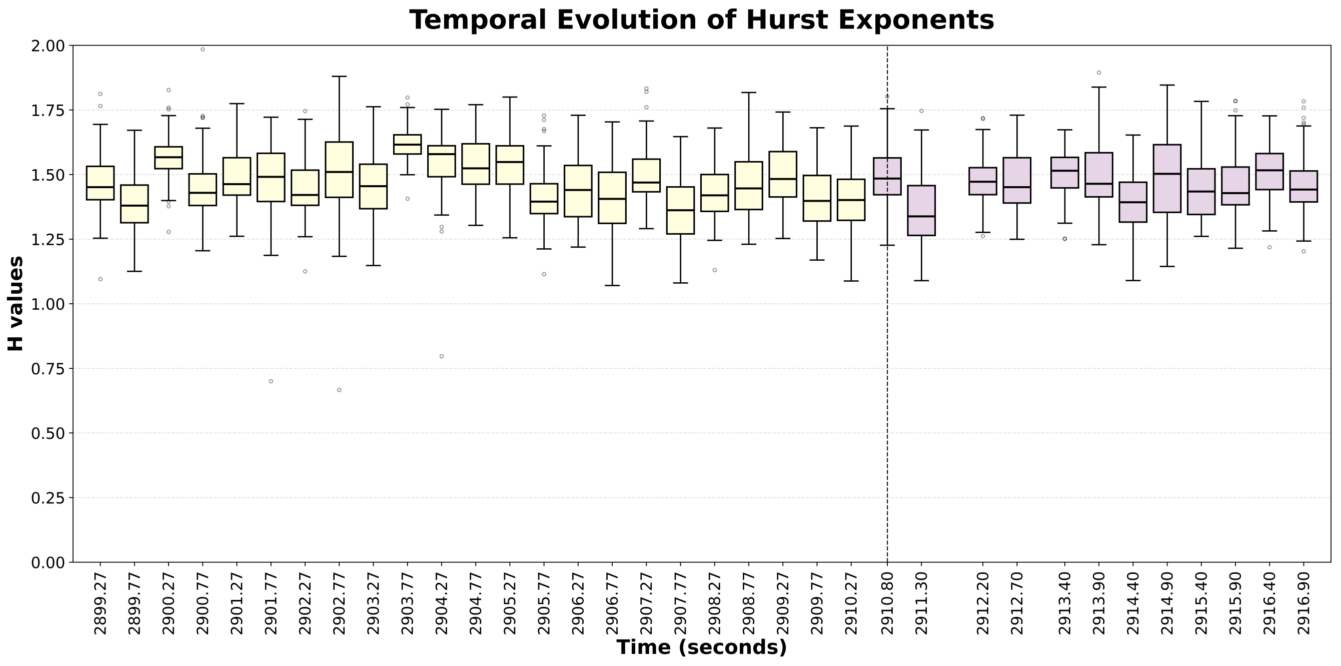

Figure S21. Temporal evolution of Hurst exponents for Patient 1, Session 6b, Trial 12 (successful trial).

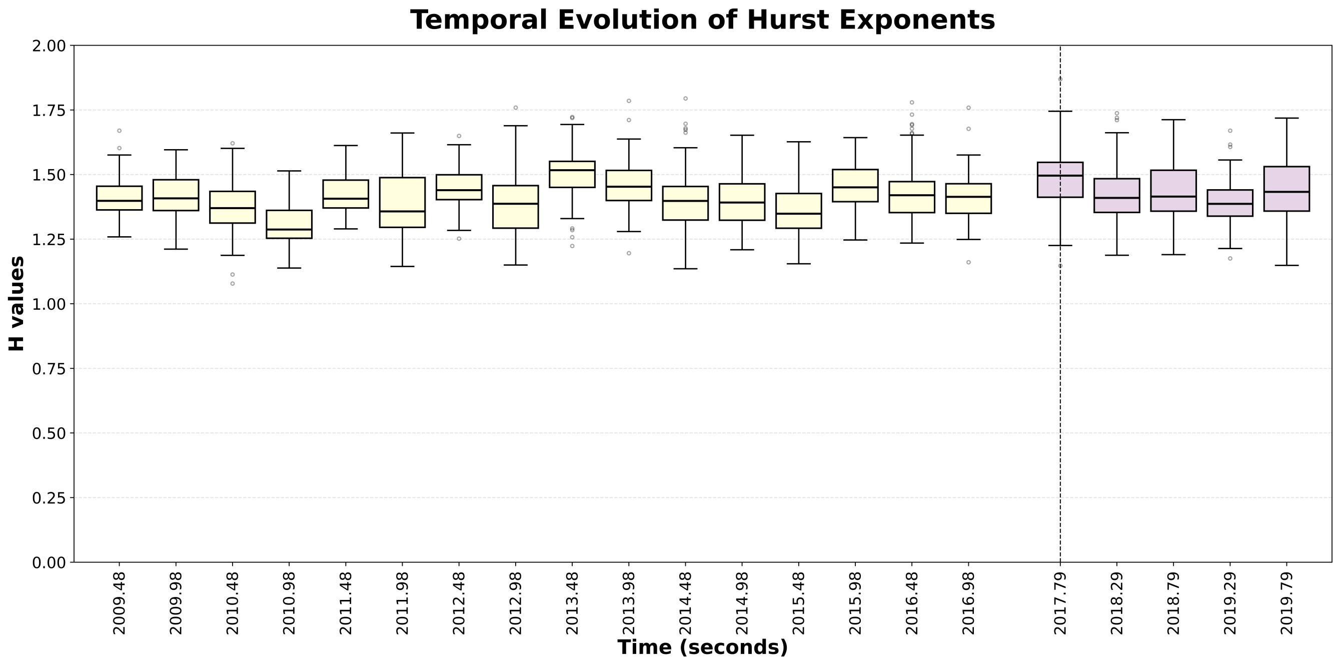

Figure S22. Temporal evolution of Hurst exponents for Patient 1, Session 8, Trial 28 (successful trial).

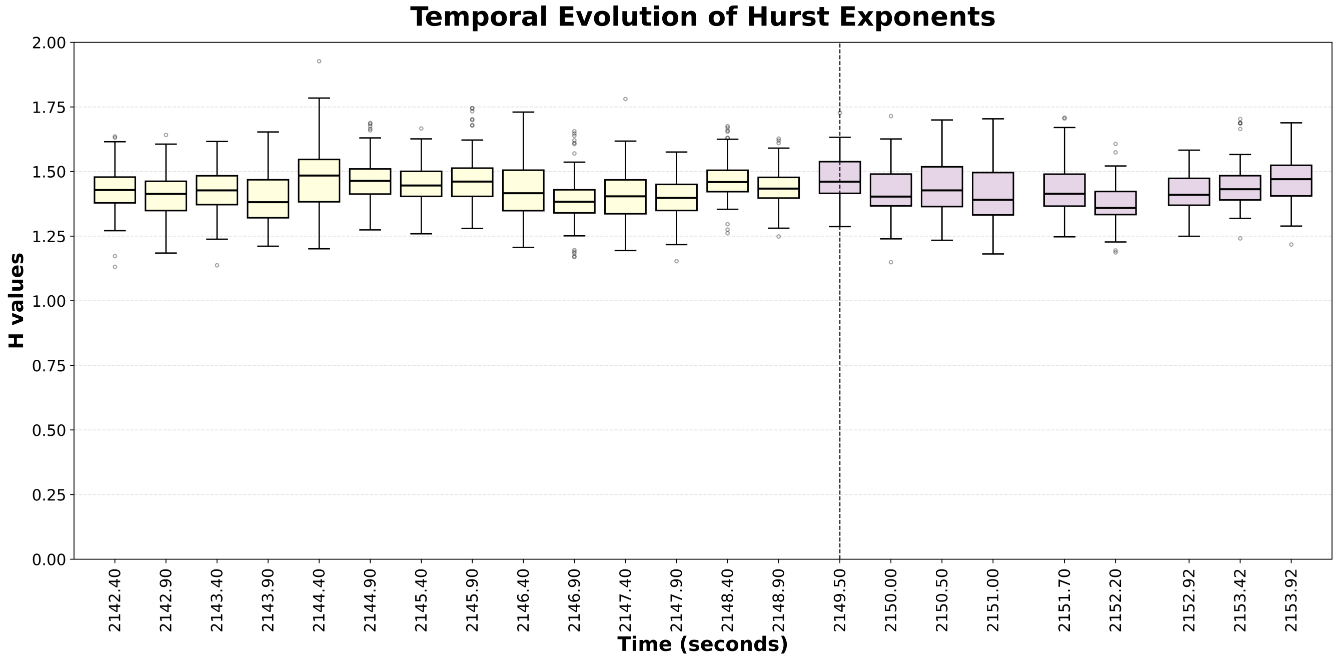

Figure S23. Temporal evolution of Hurst exponents for Patient 1, Session 8, Trial 31 (successful trial).

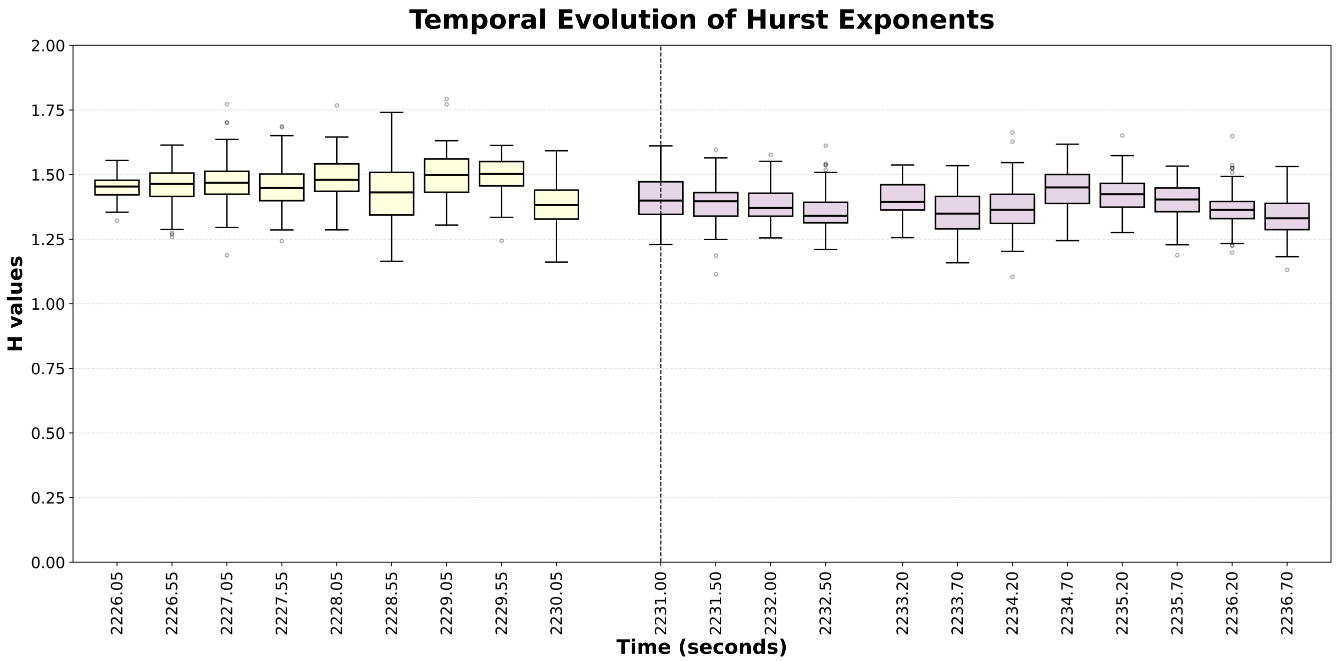

Figure S24. Temporal evolution of Hurst exponents for Patient 1, Session 8, Trial 32 (successful trial).

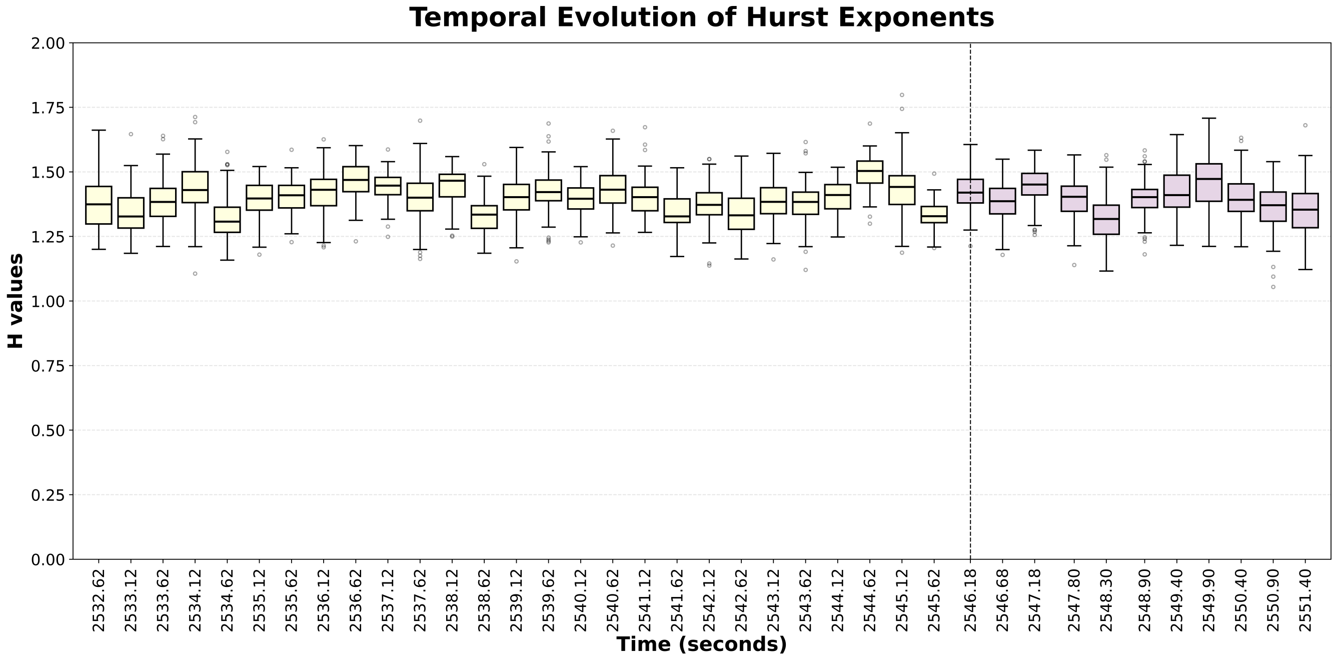

Figure S25. Temporal evolution of Hurst exponents for Patient 1, Session 8, Trial 37 (successful trial).

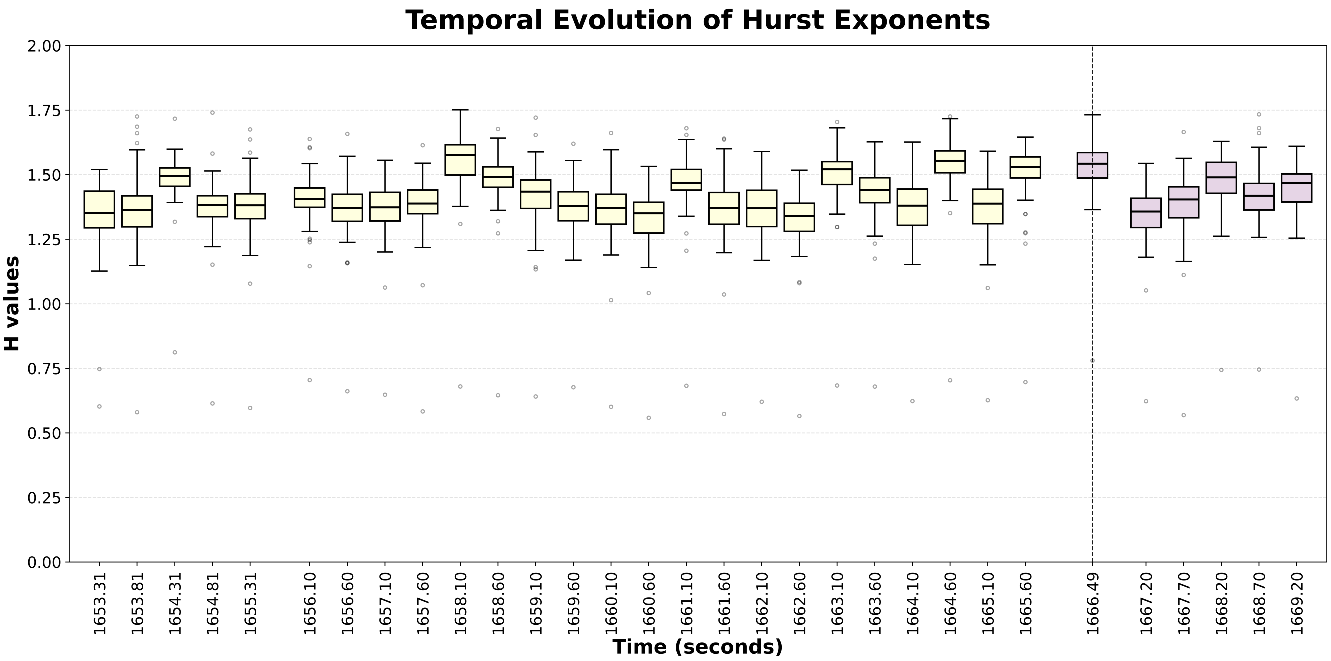

Figure S26. Temporal evolution of Hurst exponents for Patient 2, Session 3, Trial 4 (successful trial).

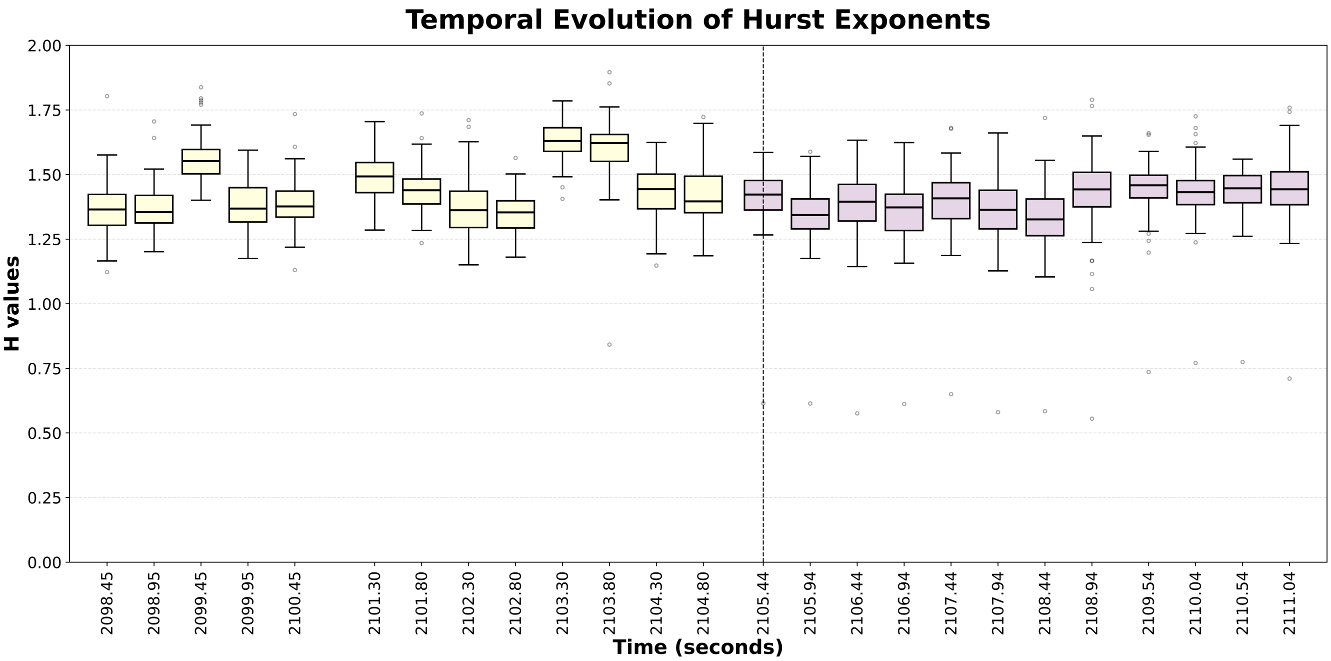

Figure S27. Temporal evolution of Hurst exponents for Patient 2, Session 3, Trial 7 (successful trial).

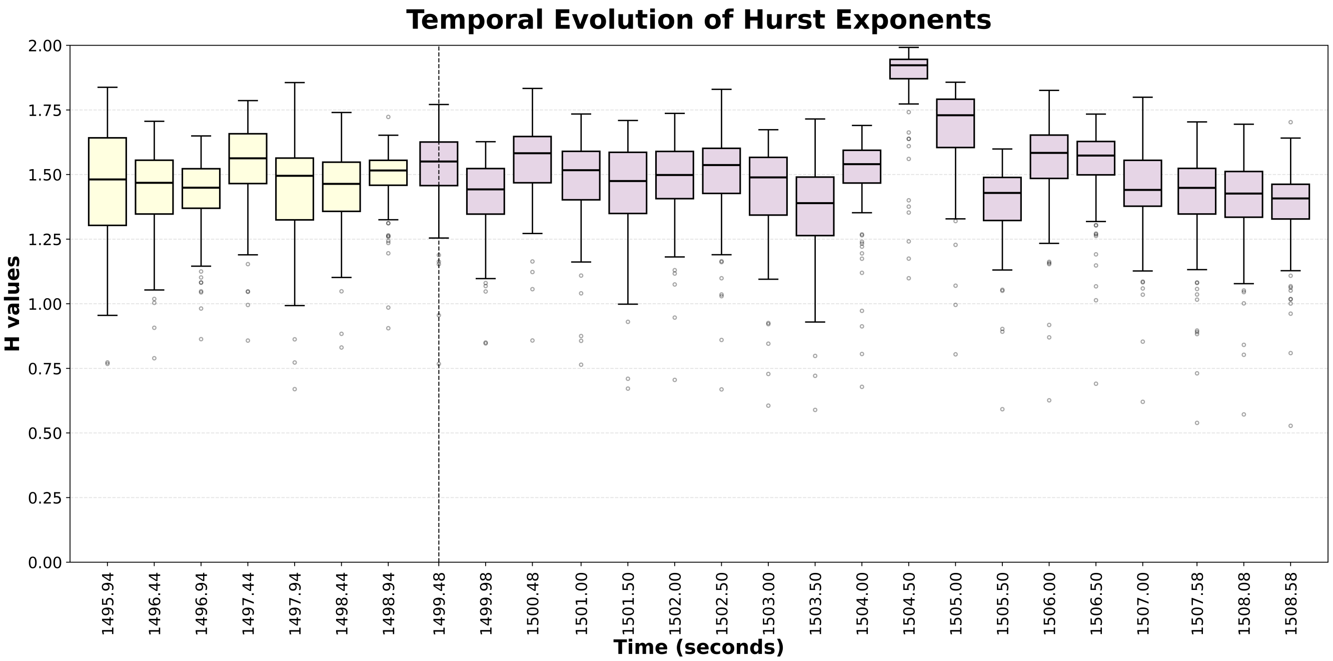

Figure S28. Temporal evolution of Hurst exponents for Patient 3, Session 2, Trial 5 (successful trial).

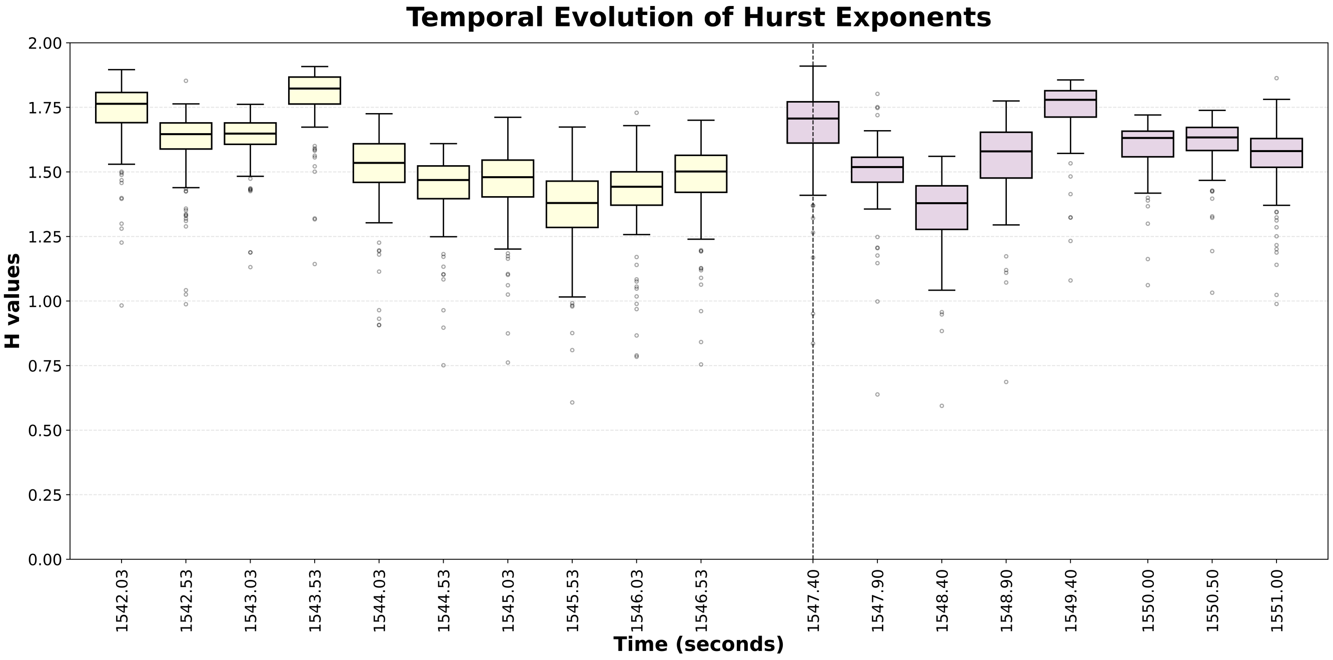

Figure S29. Temporal evolution of Hurst exponents for Patient 3, Session 2, Trial 6 (successful trial).

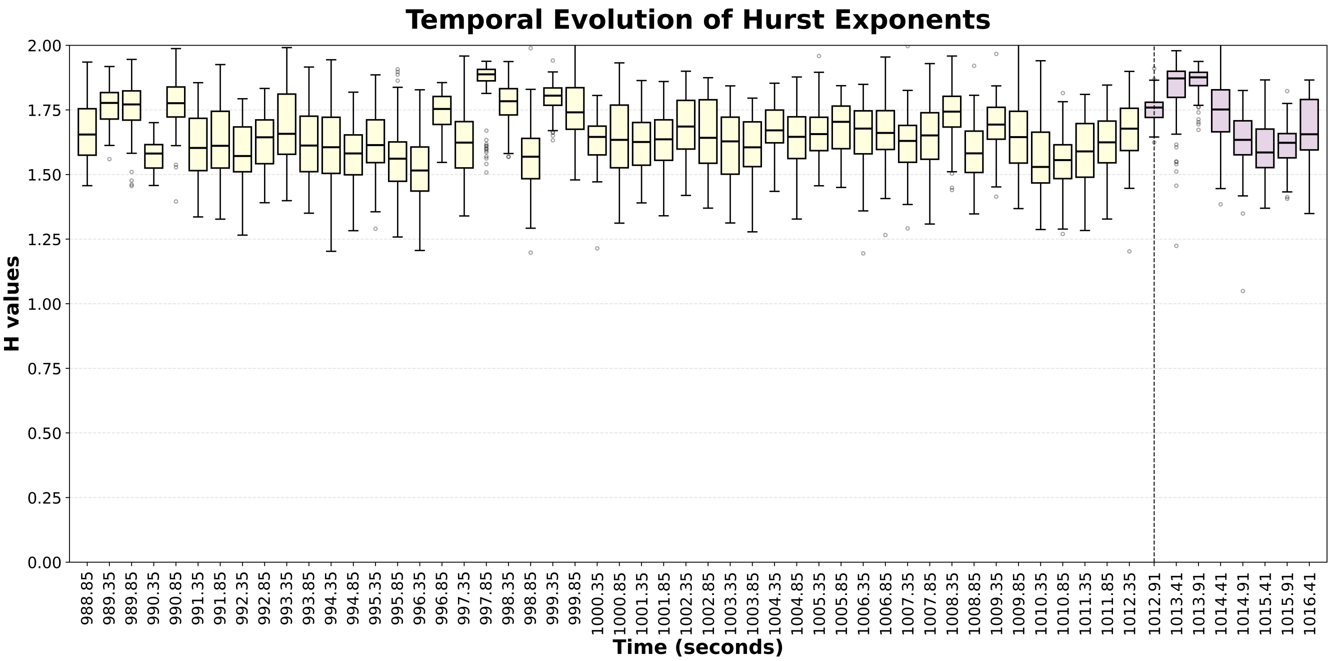

Figure S30. Temporal evolution of Hurst exponents for Patient 5, Session 2, Trial 2 (successful trial).

1.3 Temporal evolution of fractional-order exponents α (successful trials)

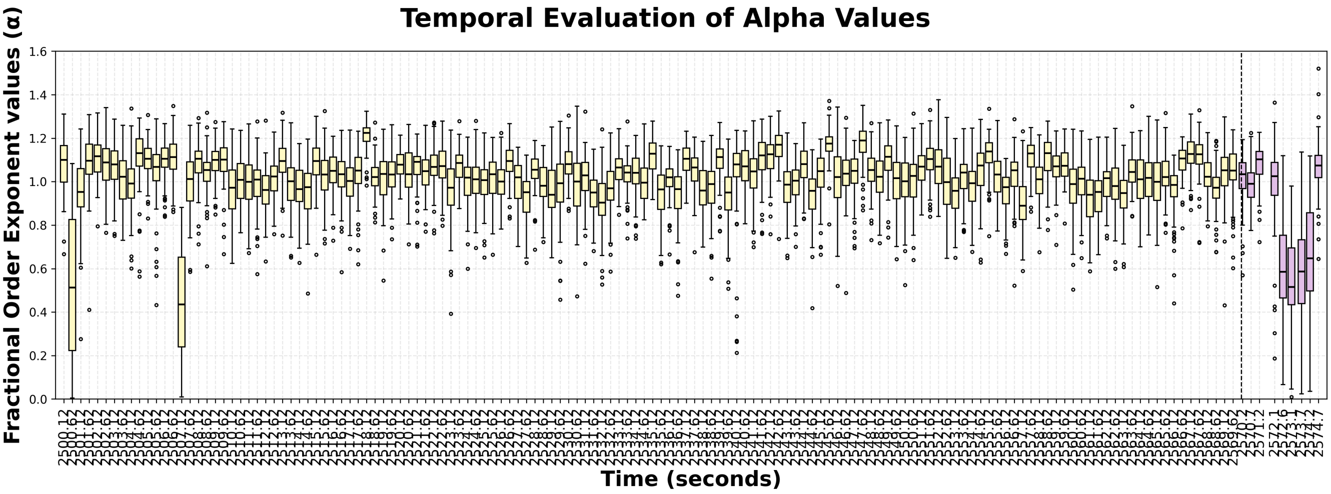

Figure S31. Temporal evolution of fractional-order exponents α for Patient 1, Session 5, Trial 1 (successful trial).

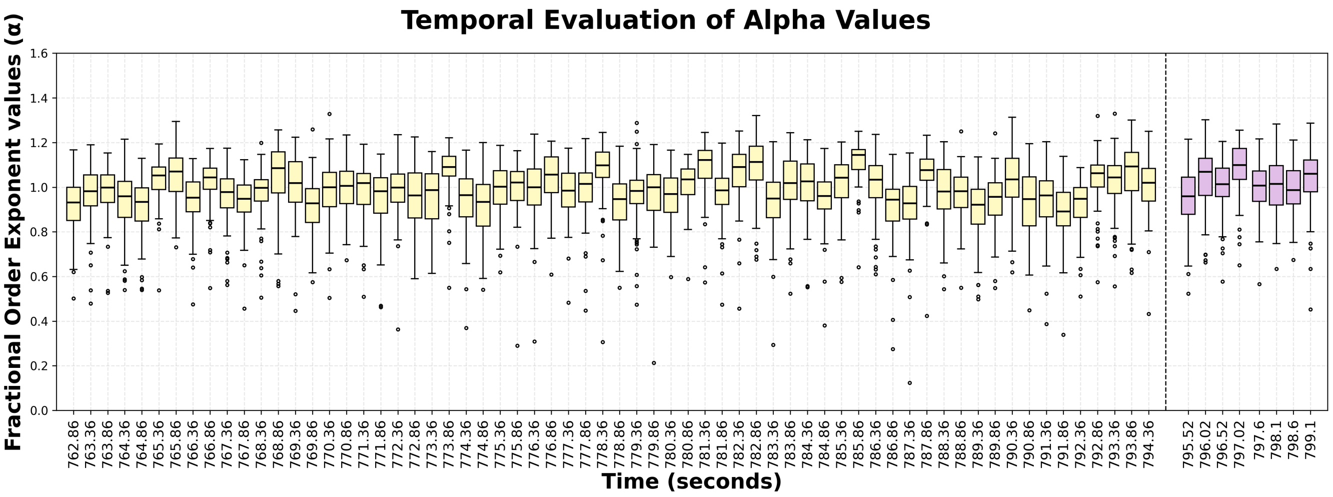

Figure S32. Temporal evolution of fractional-order exponents α for Patient 1, Session 6a, Trial 4 (successful trial).

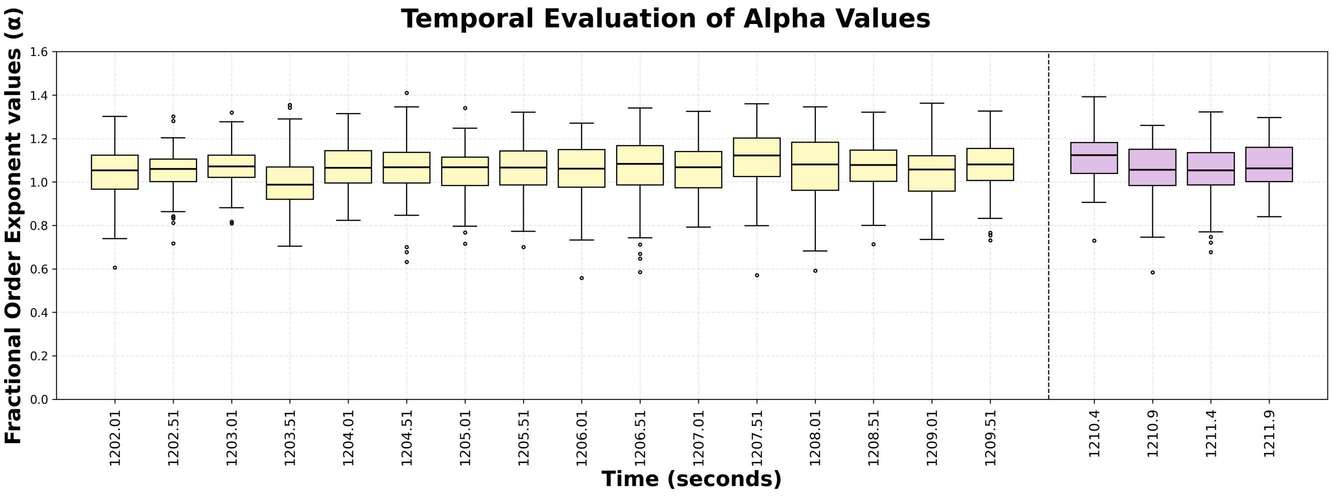

Figure S33. Temporal evolution of fractional-order exponents α for Patient 1, Session 6a, Trial 5 (successful trial).

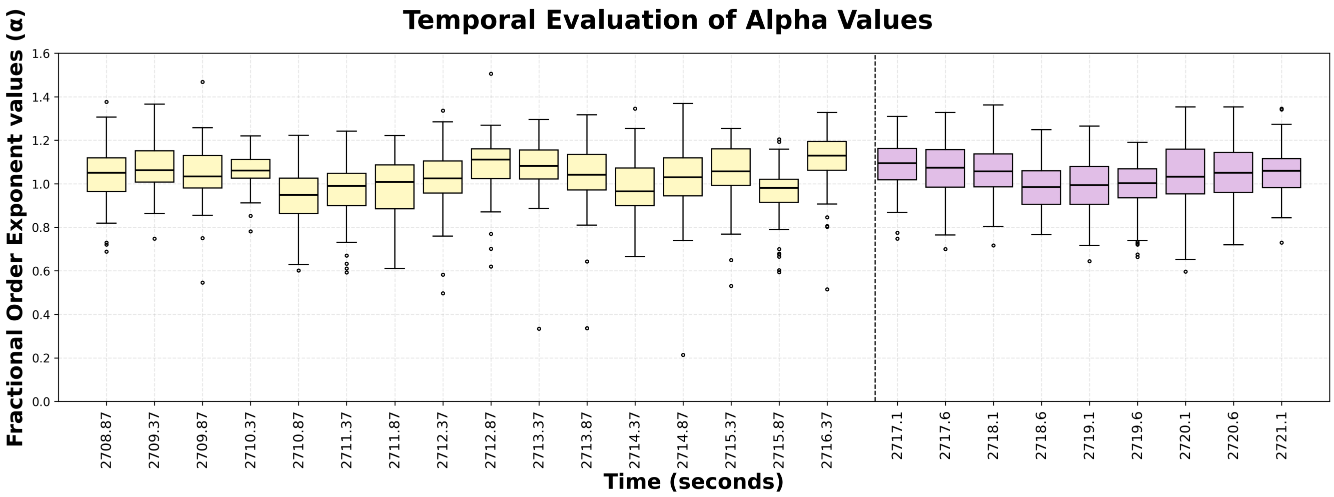

Figure S34. Temporal evolution of fractional-order exponents α for Patient 1, Session 6b, Trial 8 (successful trial).

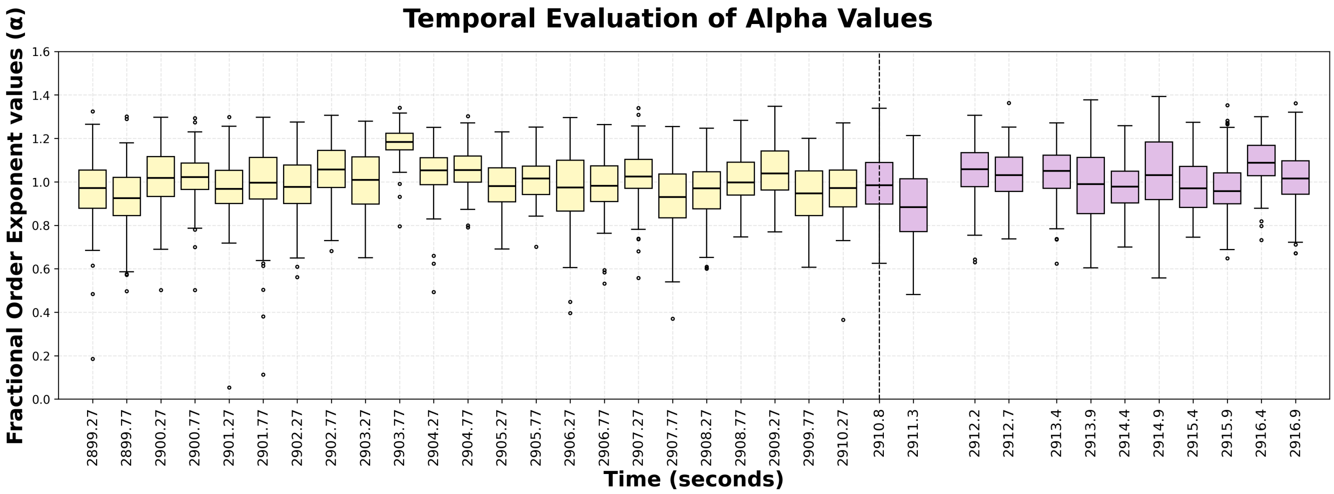

Figure S35. Temporal evolution of fractional-order exponents α for Patient 1, Session 6b, Trial 12 (successful trial).

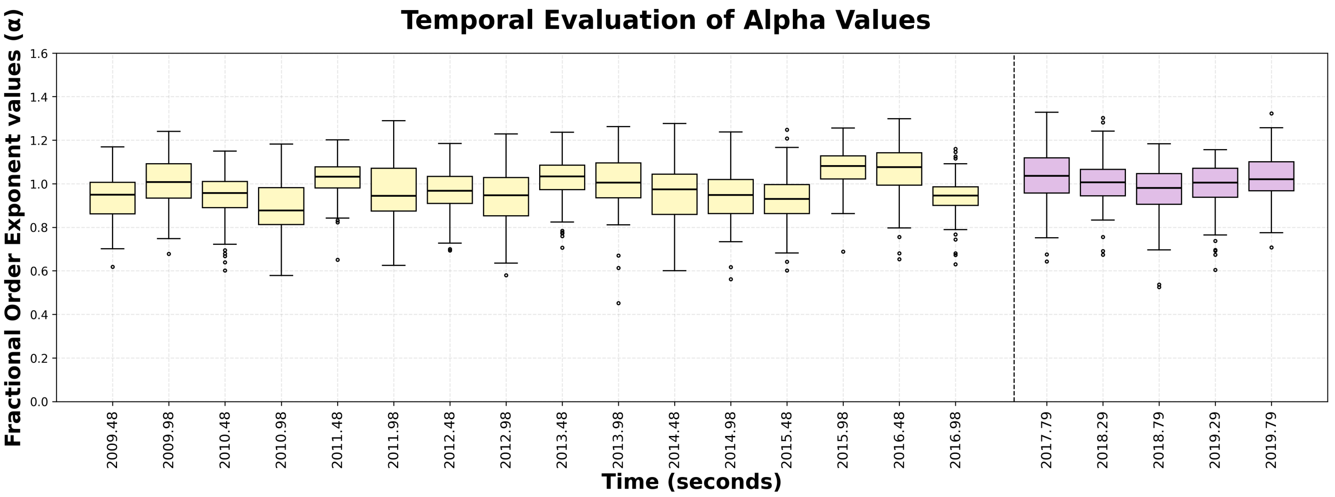

Figure S36. Temporal evolution of fractional-order exponents α for Patient 1, Session 8, Trial 28 (successful trial).

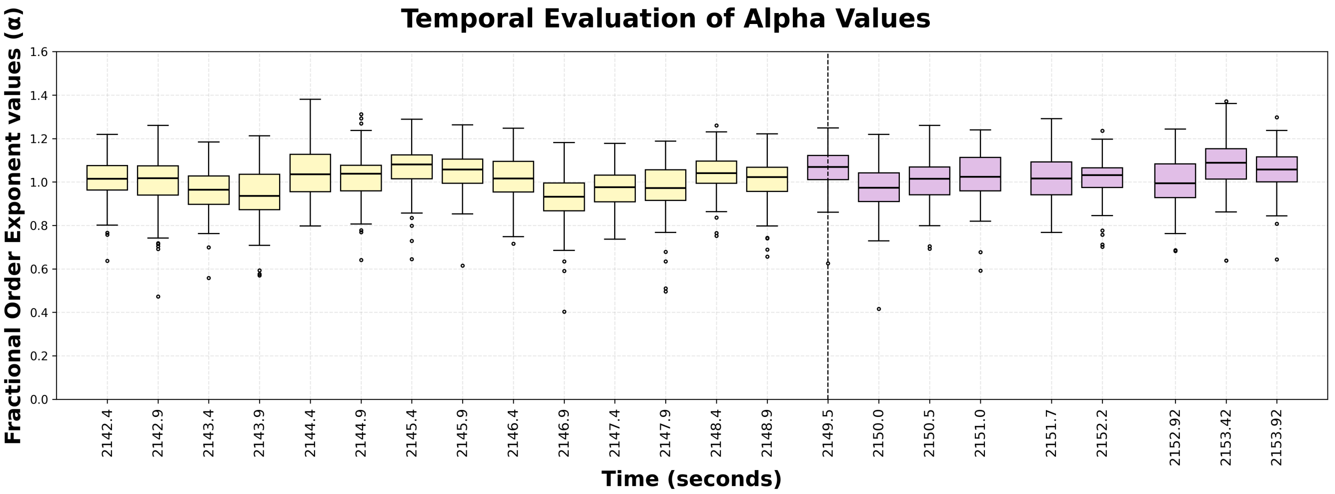

Figure S37. Temporal evolution of fractional-order exponents α for Patient 1, Session 8, Trial 31 (successful trial).

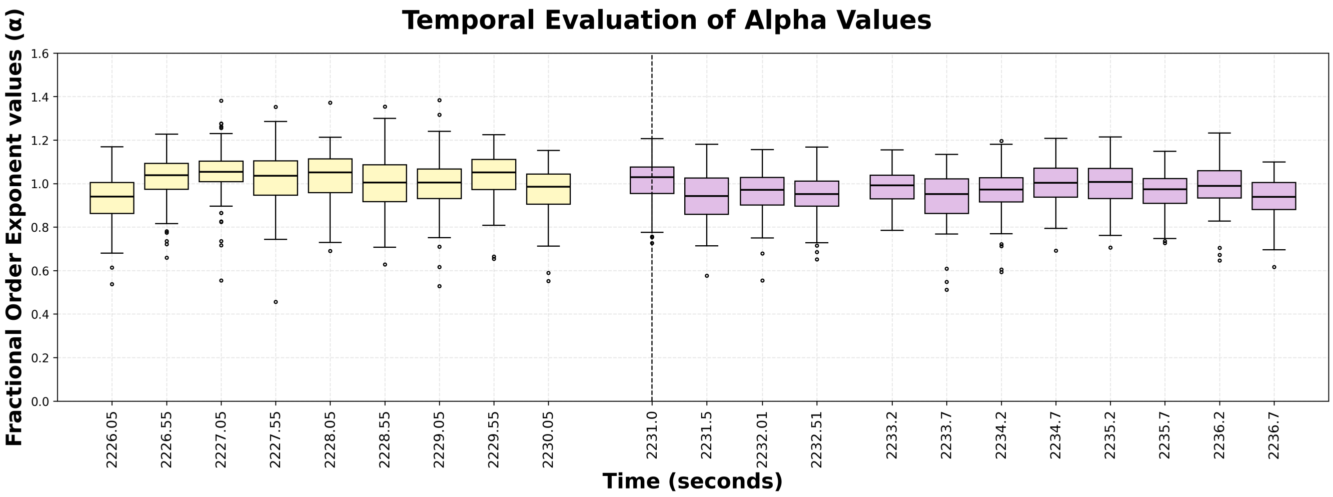

Figure S38. Temporal evolution of fractional-order exponents α for Patient 1, Session 8, Trial 32 (successful trial).

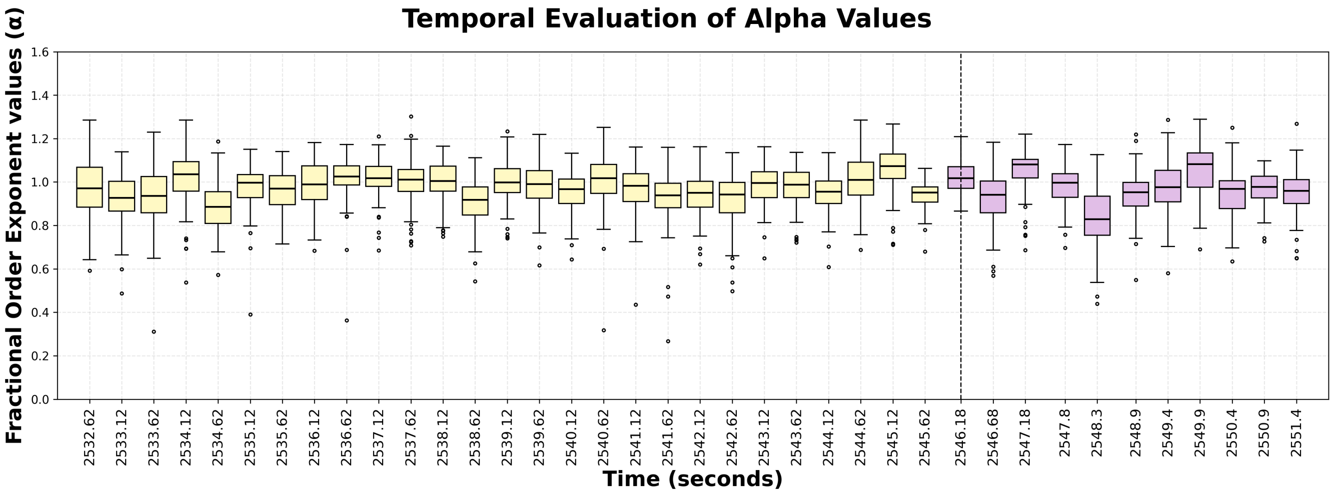

Figure S39. Temporal evolution of fractional-order exponents α for Patient 1, Session 8, Trial 37 (successful trial).

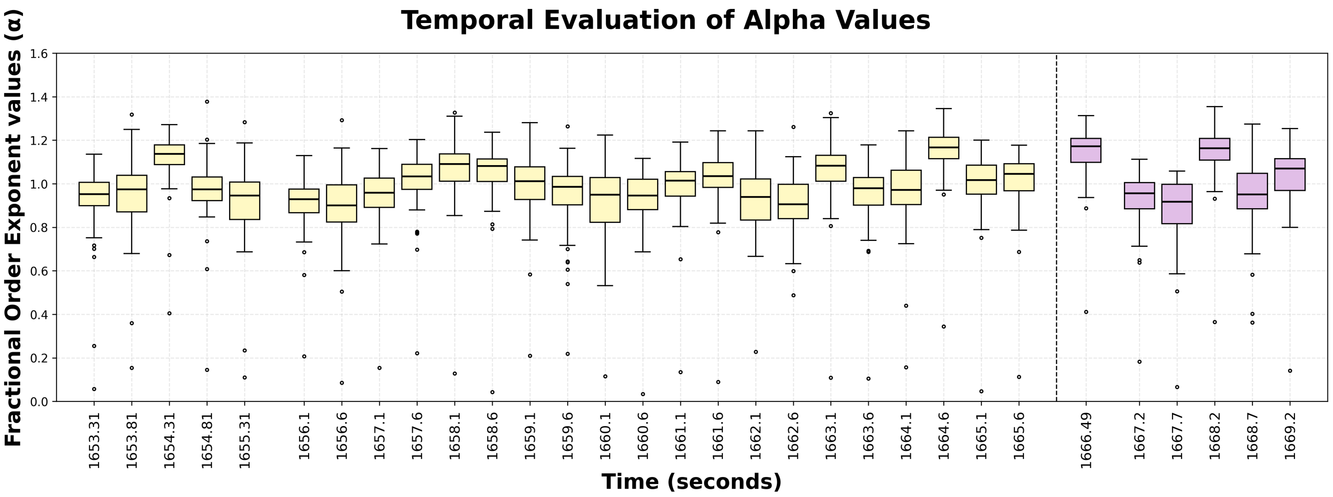

Figure S40. Temporal evolution of fractional-order exponents α for Patient 2, Session 3, Trial 4 (successful trial).

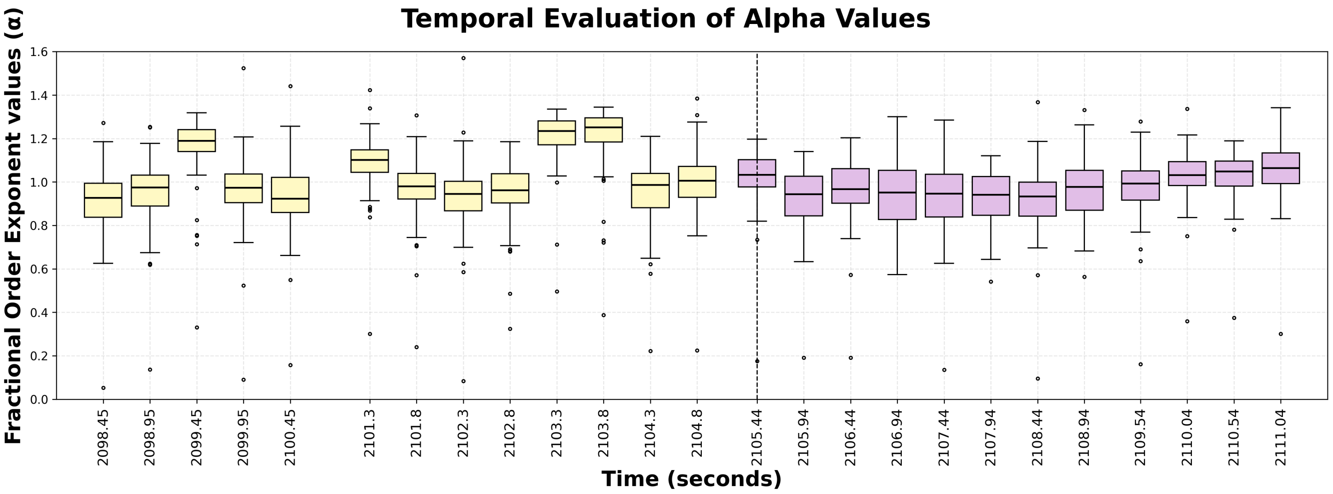

Figure S41. Temporal evolution of fractional-order exponents α for Patient 2, Session 3, Trial 7 (successful trial).

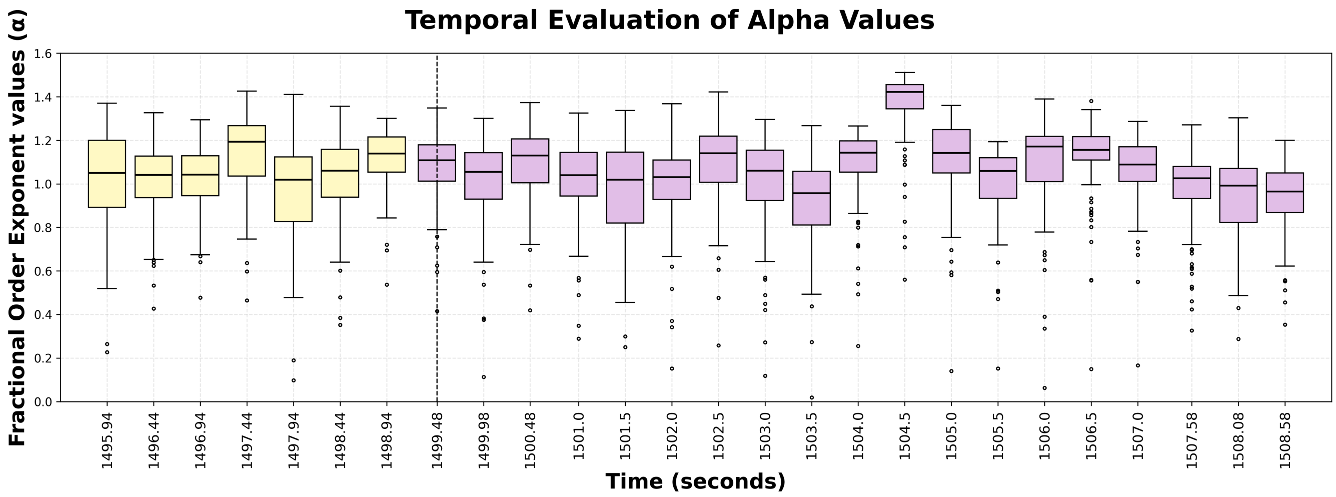

Figure S42. Temporal evolution of fractional-order exponents α for Patient 3, Session 2, Trial 5 (successful trial).

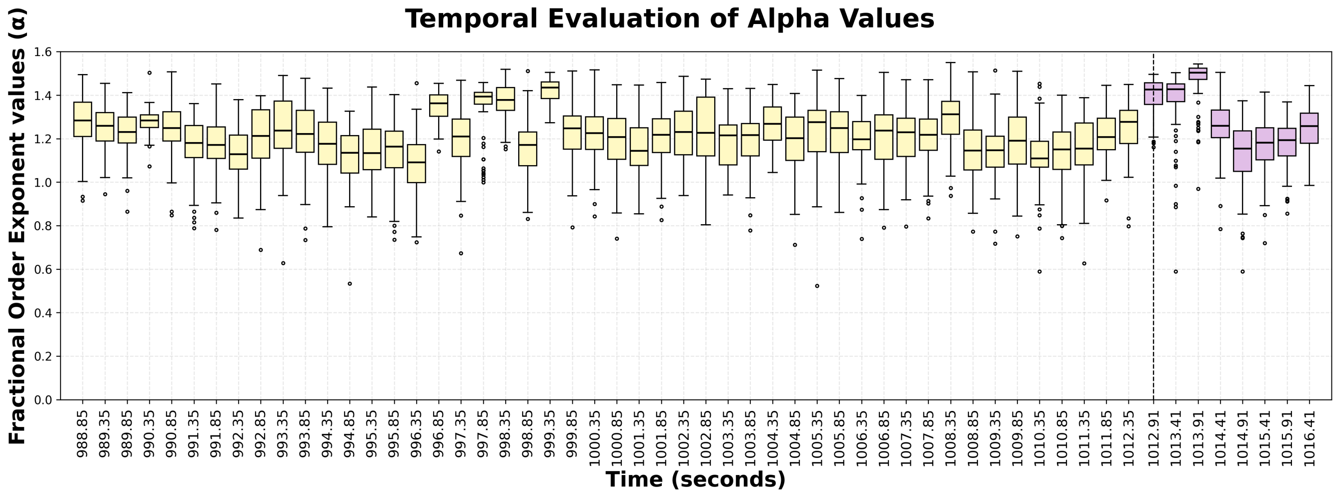

Figure S43. Temporal evolution of fractional-order exponents α for Patient 5, Session 2, Trial 2 (successful trial).
